# ^1^H NMR Quantification of Aromatic Monomers from Reductive Catalytic Fractionation

**DOI:** 10.1021/acssuschemeng.5c10351

**Published:** 2026-02-16

**Authors:** Jacob K. Kenny, Sierra Schlussel, Alexander F. Benson, Sean P. Woodworth, Hannah M. Alt, Yuriy Román-Leshkov, Gregg T. Beckham

**Affiliations:** † Renewable Resources and Enabling Sciences Center, 53405 National Laboratory of the Rockies, 15013 Denver W. Pkwy., Golden, Colorado 80401, United States; ‡ Center for Bioenergy Innovation, Oak Ridge National Laboratory, Oak Ridge, Tennessee 37830, United States; § Department of Chemical Engineering, 2167 Massachusetts Institute of Technology, 25 Ames Street, Cambridge, Massachusetts 02139, United States

**Keywords:** Proton nuclear magnetic resonance, lignin monomers, quantification, model compound, reductive catalytic fractionation

## Abstract

Reductive catalytic fractionation (RCF) can produce high yields of aromatic monomers from lignin in native biomass. Quantification of these aromatic monomers is a well-known but demanding task, in part due to the lack of commercially available standards. Here, we demonstrate ^1^H NMR spectroscopy as a complementary method to rapidly quantify aromatic monomer concentrations in RCF oils. The method exhibited good agreement with measurements from ultrahigh pressure liquid chromatography (UHPLC) for 96 RCF oils with varying monomer selectivity, with average absolute deviations of individual monomer yields between 0.5 and 1.1 wt % (relative 11–17%) and R^2^ values above 0.9 compared to conventional UHPLC quantification. Quantification of S-type monomers, including for 4-ethylsyringol and 4-propenylsyringol, was generally reliable. The validity of G-type monomer quantifications depended on reaction selectivity due to overlap between peaks of 4-ethylguaiacol and 4-(3-hydroxypropyl)-guaiacol. The method could be applied on crude RCF oils without needing to perform the liquid–liquid extraction typically done for RCF reactions, thereby providing a convenient way to quantify lignin extraction and aromatic monomer yield. Overall, ^1^H NMR spectroscopy can serve as a rapid primary quantification or secondary validation method for RCF monomer yield and selectivity measurements.

## Graphical abstract

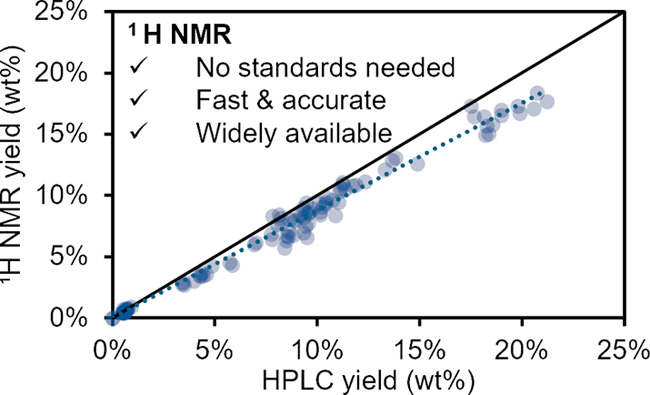

## Introduction

Reductive catalytic fractionation (RCF) is a lignin-first biorefining approach that simultaneously extracts and catalytically converts lignin into 4-substituted phenolic monomers.^1^ Depending on the biomass substrate, the yield of aromatic monomers can reach ∼ 40 wt %, making RCF a promising technology for biomass conversion with applications in fuels and chemicals.^2–4^ The most common aromatic monomers are 4-propyl- and 4-(3-hydroxypropyl)-substituted syringol and guaiacol. Reaction conditions can also be tuned to produce additional monomers, such as 4-ethyl and 4-propenyl-substituted compounds.^5–10^ Other aromatic products include 4-allyl-substituted products, 4-allylsyringol and eugenol,^11^ the monolignols coniferyl and sinapyl alcohol,^12^ and etherified products such as 4-(3-methoxypropyl)-syringol/guaiacol,^7,13^ but the yields of these products are usually low in conventional RCF reactions. Depending on the substrate, additional aromatic monomers may also be produced, such as *p*-hydroxybenzoate, or *p*-coumaric and ferulic acid derivatives, further increasing the variety of products that may be observed from RCF reactions.^14^

Accurate, unambiguous quantification of the individual monomers from RCF reactions is essential for connecting reaction parameters to RCF process performance. Quantification of these monomer products is often performed using gas chromatography with detection via flame ionization detection (GC-FID) or with mass spectrometry (GC-MS).^1,13,14,16^ High performance liquid chromatography (HPLC) has also proven suitable for quantification in a way that is more tolerant of aqueous solvent compositions relative to GC.^15^ Although quantification of RCF monomers is routine, substantial effort must be made to ensure proper quantification. First, many of the monomers, especially the S-type derivatives, are not widely commercially available for use as analytical standards and therefore must be synthesized in-house or purchased from specialty synthesis companies.^13^ In our experience, 4-propenylsyringol (PES) has proven especially difficult to synthesize or procure as it is prone to degradation. Approximate methods such as effective carbon number (ECN) have been used in lieu of authentic standards, but this comes at the cost of accuracy.^1,16^ Once standards are obtained, chromatographic methods must be developed to resolve the 8–18 monomers typically found in RCF oils, depending on the biomass substrate. Stock solutions of the standard compounds must be maintained for recurring calibration, as both column and detector behavior, and therefore response factors, can change over time.^1^ Conversely to chromatographic methods, nuclear magnetic resonance (NMR) spectroscopy is a common tool available at many institutions. We recently reported quantification of RCF oil yields via ^1^H NMR spectroscopy as an alternative to gravimetric weighing as part of a high-throughput RCF pipeline,^17^ and in the current work, we extend this approach to the quantification of individual aromatic monomers in RCF oils.

## Results and Discussion

### Identifying Aromatic Monomers from the ^1^H NMR Spectrum of RCF Oil

The use of NMR spectroscopy for quantification requires that resonances from the compounds of interest be unambiguously assigned in the NMR spectrum. To evaluate ^1^H NMR as a monomer quantification method, we first compared the ^1^H NMR spectra of poplar RCF oil generated under typical RCF conditions (Ru/C, 200 °C, methanol, 30 bar H_2_, 3 h after a 30 min heating ramp; Figure 1A) with spectra of common phenolic monomers, namely 4-propyl, 4-propenyl, 4-ethyl, and 4-(3-hydroxypropyl)-substituted syringol and guaiacol (Figure 1B, Figures S1–S20; model compounds were either purchased or synthesized in-house).^13^ The ^1^H NMR experiment was performed on the dried RCF oil without performing liquid–liquid extraction, thereby avoiding the time-consuming workup of filtration, liquid–liquid extraction, and evaporation which is typically done for RCF reactions.^17^ The ^1^H NMR experiment was performed with 32 scans and a recycle delay (d1) of 3 s, leading to a total experiment time of ∼6 min; other NMR experimental details and development are detailed in the Supporting Information.

**Figure 1:**
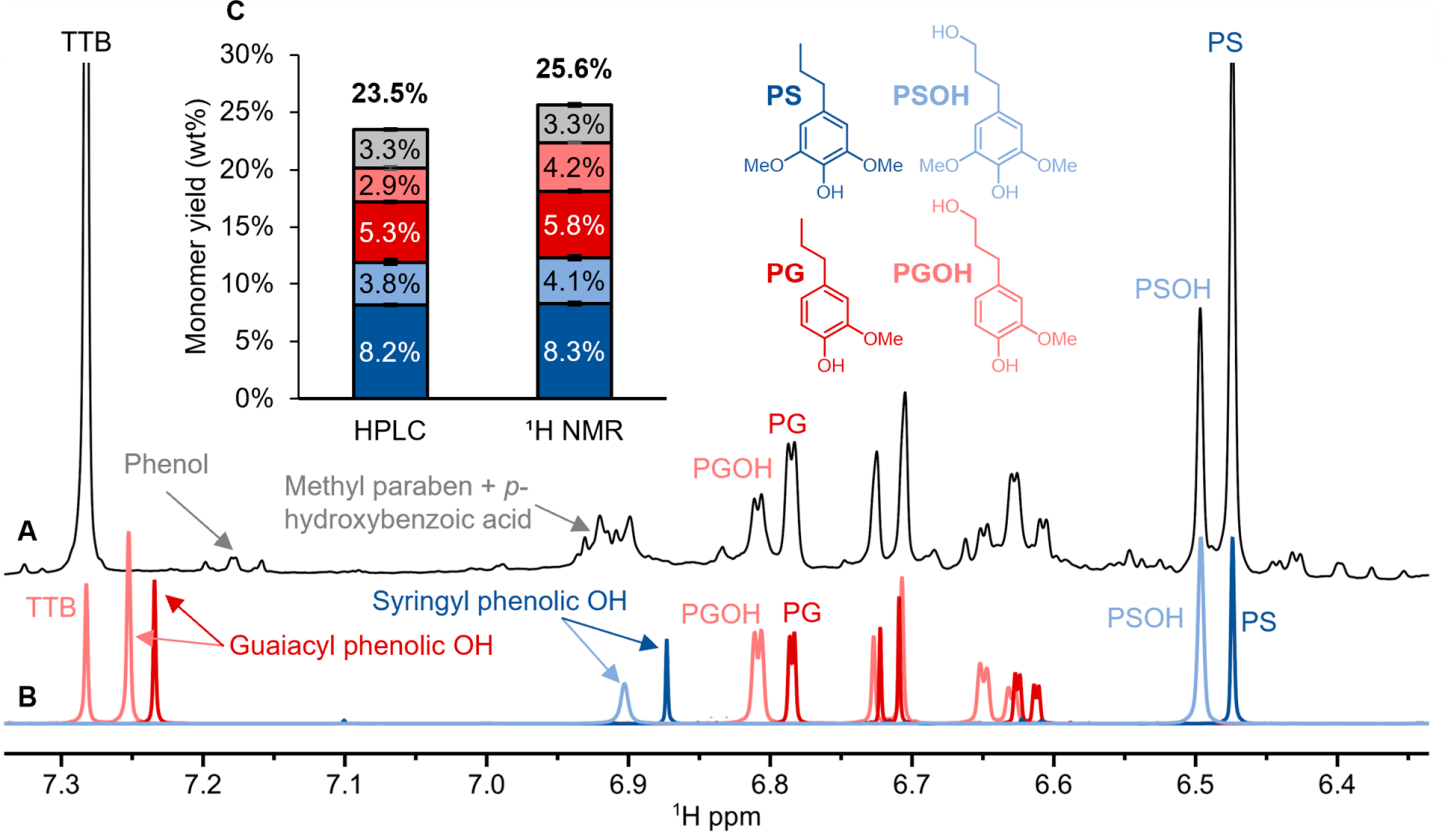
^**1**^H NMR spectrum of RCF oil produced using Ru/C, leading to high selectivity in 4-propyl monomers with minor contributions from 4-(3-hydroxypropyl) monomers. (A) ^1^H NMR spectrum of the RCF oil with peaks labeled. (B) ^1^H NMR spectrum of authentic standards. (C) Comparison of monomer quantifications obtained using UHPLC and ^1^H NMR spectroscopy. RCF conditions: 2 g poplar, 400 mg Ru/C (5 wt % Ru), 200 °C, 30 bar H_2,_ 3 h. The reaction product was filtered directly from the reactor and liquid–liquid extraction was not performed prior to NMR analysis. NMR specifications: 0.5 mL of reaction filtered liquor dried under flowing N_2_ and dissolved in 1 mL 10:1 acetone-d_6_/methanol-d_4_ with 1 mg/mL 1,3,5-tri*-tert*-butylbenzene (TTB) as the internal standard. The methanol-d_4_ was required to fully solubilize the unextracted sample. 32 scans, 3 s delay (d1), 0.3 Hz line broadening, and one level of zero-filling.

The aromatic region of the ^1^H NMR spectrum of the RCF oil displayed prominent peaks corresponding to the four primary monomers. Distinct singlet resonances in the range 6.46–6.50 ppm were present, corresponding to the aromatic S_2/6_ protons of 4-propylsyringol (PS) and 4-(3-hydroxypropyl)-syringol (PSOH). Aromatic resonances of G-type monomers were more complex due to the presence of three unique protons on the aromatic ring, but the G_2_ peaks of 4-propylguaiacol (PG) and 4-(3-hydroxypropyl)-guaiacol (PGOH) were clearly observable in the range of 6.77–6.82 ppm. Although also clearly visible in the NMR spectrum, the G_5_ and G_6_ resonances of these two monomers overlapped substantially due to their large coupling constants (∼8 Hz) and therefore were not expected to be reliable for quantification.

As noted in our previous work, resonances attributable to the monomers *p*-hydroxybenzoic acid and methyl paraben were also observable at 7.87–7.93 ppm (H_2/6_) and 6.89–6.94 ppm (H_3/5_; Figure 2A, Figures S9, S10). Phenol, which arises from *p*-hydroxybenzoic acid decarboxylation, is also present in this oil but has a more complicated ^1^H NMR spectrum (Figure S11) with resonances at 7.14–7.22 ppm (2H, m) and 6.77–6.85 ppm (1H, m, 2H, m). The latter resonances overlapped with the G_2_ proton resonances; high phenol content may complicate or preclude the use of this G_2_ resonance for quantification of the G-type monomers (*vide infra*).

**Figure 2:**
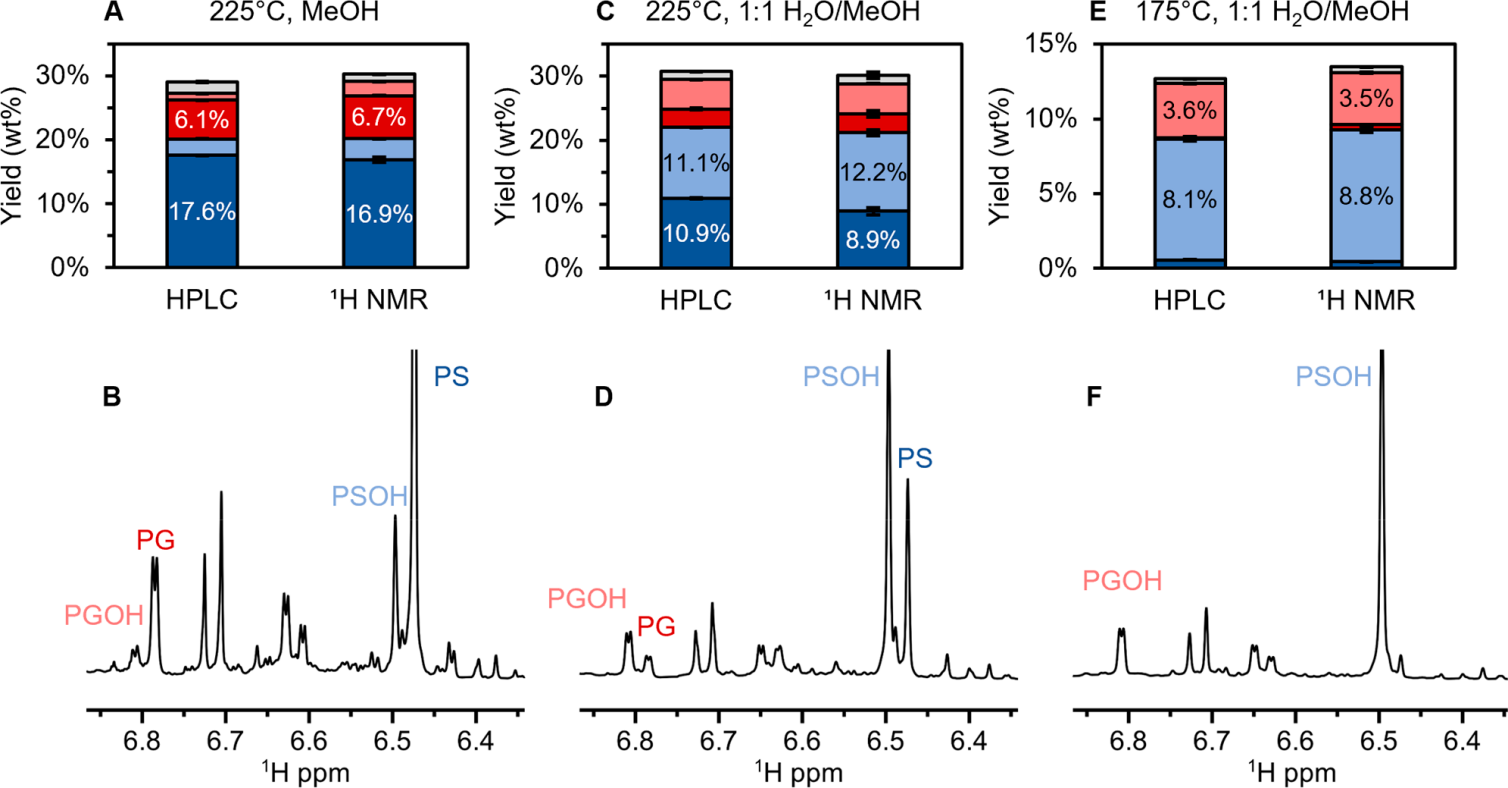
Comparison of monomer quantifications obtained from UHPLC and ^1^H NMR for reactions with alternative conditions. All reactions were performed on 2 g of poplar with 400 mg of Ru/C and 30 bar H_2_, and liquid–liquid extraction with ethyl acetate was performed to isolate the lignin-derived products to obtain the oil yield. (A, B) 225 °C, 3 h. (C, D) 225 °C, 0.5 h. (E, F) 175 °C, 0.5 h. NMR specifications were as previously stated.

Although typically present in lower amounts compared, 4-ethyl and 4-propenyl-substituted monomers can also be produced during RCF depending on the reaction conditions.^9,13^ NMR spectra of 4-ethylsyringol (ES, S_2/6_: 6.49 ppm) and PES (S_2/6_: 6.66 ppm) indicate they should be distinguishable in RCF oils by their aromatic resonances (Figures S2, S4). Given the proximity of the ES resonance to those of PS and PSOH, quantification of ES may be difficult at the low concentrations typically present in RCF oil. Importantly, both minor G-type products displayed some degree of overlap with the primary products PG and PGOH. The G_5_ of isoeugenol (PEG), which appeared as a broad doublet of doublets, partially overlapped both with the G_2_ resonance of PG. PEG could still be identified from its G_2_ resonance at 6.99 ppm, thereby also providing a way correct the PG integral by the PEG amount if needed. The G_2_ resonance of 4-ethylguaiacol (EG, 6.80 ppm) partially overlapped with the G_2_ of PGOH. This overlap prevents the unambiguous determination of EG in RCF oil by its aromatic resonance, as well as decreases confidence in the quantification of PGOH from its G_2_ resonance. Additional information was therefore needed to confidently determine the amount of EG and PGOH monomers in RCF oils.

In contrast to the aromatic region, resonances in the aliphatic region were more differentiated based on the side-chain identity rather than syringyl or guaiacyl monomer type, thus providing an additional way to corroborate selectivity (Figure S21). In the RCF oil spectrum, the region attributed to the β protons of ethyl side chains (1.13–1.20 ppm) displayed low signal (Figures S22, S23), confirming that ES/EG abundance was low. In contrast, resonances corresponding to the γ and β protons of the 4-propyl side chains of PG and PS were clearly visible in the range of 0.86–0.93 ppm and 1.53–1.64 ppm, respectively. Clear resonances attributable to PSOH and PGOH were also observed, however, model compound spectra indicated that these may overlap slightly with other compounds: the α with the α resonance of ES/EG and β with the γ resonance of PES/PEG (Figure S21B). Only the γ resonances of PSOH/PGOH in the range 3.45–3.58 ppm were clearly separated from resonances of the other model compounds tested. Although their content was low in this oil, model compound spectra indicate that PES and PEG alkene resonances located at 6.28–6.35 ppm and 6.06–6.15 ppm corresponding to the α and β protons, respectively. These resonances could therefore also be diagnostic of the presence of unsaturated compounds such as these. Taken together, these features show that the aliphatic region provides insight into the side-chain selectivity in the RCF reaction and can be used to confidently assign resonances in the G-type region.

### Comparing Yields from UHPLC and ^1^H NMR

To test the ability to quantify individual aromatic monomers from the ^1^H NMR spectrum, resonances in the aromatic region were integrated relative to the internal standard 1,3,5-tri*tert*-butylbenzene (TTB) (Figure 1C). The total monomer yield measured by UHPLC was 23.5 ± 0.6 wt %, (error bars indicate the range of duplicate RCF experiments), with high selectivity to PS and PG (57 ± 1 wt %, expressed on the basis of total monomers including *p*-hydroxybenzoic acid and methyl paraben), and PSOH and PGOH (29 ± 1 wt %), Figure 1C).^18^ The yields of lignin-derived phenolic monomers from ^1^H NMR closely match those from UHPLC. PS (UHPLC 8.2 ± 0.1 wt %, NMR 8.3 ± 0.1) and PSOH (UHPLC 3.8 ± 0.3 wt %, NMR 4.1 ± 0.2 wt %) were quantified most closely, but PG (UHPLC 5.26 ± 0.07 wt %, NMR 5.8 ± 0.1) and PGOH (UHPLC 2.9 ± 0.2 wt %, NMR 4.2 ± 0.1 wt %) monomers were slightly overquantified (Figure 1C). Integration of the S_2/6_ of PES gave a yield of 0.40 ± 0.01 wt % versus 0.79 ± 0.03 wt % from UHPLC. Similarly, integration of the PEG G_2_ gave a yield of 0.60 ± 0.03 wt %, compared to 0.22 ± 0.01 wt % quantified via UHPLC. These discrepancies indicate that the NMR method may not be capable of quantifying low-yield monomers (*vide infra*). To correct for the overlap of PG with PEG, 50% of the PEG G_2_ peak integral value was subtracted from the PG G_2_ integral used for quantification. Note that phenol was not included in this particular UHPLC analysis and thus was omitted from the NMR quantifications.

The aliphatic region indicates that the presence of minor monomers (e.g., EG, PEG) is unlikely to cause overestimation of PGOH and PG in this oil. However, RCF oil also contains a substantial amount of dimers and oligomers.^19,20^ In contrast to GC and UHPLC, which separate compounds based on volatility or intermolecular interactions, NMR chemical shifts reflect the local chemical environment. Side-chain functionalities of higher molar mass species often resemble those of monomers, making overlap between monomer and oligomer resonances possible. Although the overlap of monomer/oligomer resonances is likely, it was not expected that meaningful conclusions could be drawn from comparison of monomer spectra with the spectra of a higher molar mass model compound, given the diversity of higher molar mass lignin compounds produced in RCF.^19,20^

Given the promising results of the initial test, we sought to validate the proposed NMR-based monomer quantification using RCF oils produced under different reaction conditions including addition of water cosolvent (up to 50 vol % H_2_O), residence times (0.5–6 h), temperatures (175–225 °C), and different poplar substrates.^21^ These experiments produced RCF oils with varying selectivities to 4-propyl and 4-(3-hydroxypropyl) products, with three examples shown in Figure 2. At 225 °C in methanol, the selectivity to PS and PG reached 81.3 ± 2%, which was slightly higher than the result at 200 °C. The corresponding ^1^H NMR spectrum showed large resonances for PS and PG (Figure 2A–B). With 50 vol % H_2_O as the cosolvent, the selectivity shifted to an approximate 50:50 split between 4-propyl and 4-(3-hydroxypropyl) products (Figure 2C–D). At 175 °C, the selectivity to PSOH and PGOH further increased to 92.4 ± 0.5 wt % (Figure 2E–F). These shifts in product distribution were also captured by the ^1^H NMR spectra, (Figure 2B, D, F). The resulting quantifications are shown in Figure 2A, C, E. The change in selectivity is clearly captured in ^1^H NMR spectrum and corresponding monomer quantifications.

In total, 96 separate RCF oils were analyzed with ^1^H NMR spectroscopy and UHPLC under varying conditions. NMR quantifications for the four primary monomers (PS, PSOH, PG, PGOH) showed good correlations with UHPLC values, with R^2^ values in the range of 0.90–0.98 (Figure 3A–D). As noted previously, slightly elevated values were measured for PGOH at low (∼ 2.5 wt %) yields. Monomers with lower selectivity and yields showed less consistent agreement between UHPLC and ^1^H NMR (Figure 3E–H), although standard quantification methods (e.g., GC-FID, UHPLC) are also be expected to be less reliable at low concentrations. At these yields, resonances were difficult to distinguish from the surrounding signals. The decision to integrate the region depended on the appearance of a distinguishable resonance and whether additional information on selectivity could be gleaned from the aliphatic region. For example, a case of low yields but identifiable resonances for PES and PEG is shown Figure S24. This led to cases where a product was quantified by one method but not the other, resulting in data points on either the x- or *y*-axis. Given the lack of identifiable signal, EG was not independently integrated but rather estimated from the ES yield and the ratio of PS/PG measured from NMR. Overall, ^1^H NMR accurately estimated monomer yields compared to UHPLC for monomers produced with high selectivity. Similar agreement between UHPLC and ^1^H NMR quantifications were obtained for switchgrass (including ferulate- and *p*-coumarate-derived products; Figure S25) and pine (Figure S26).

**Figure 3:**
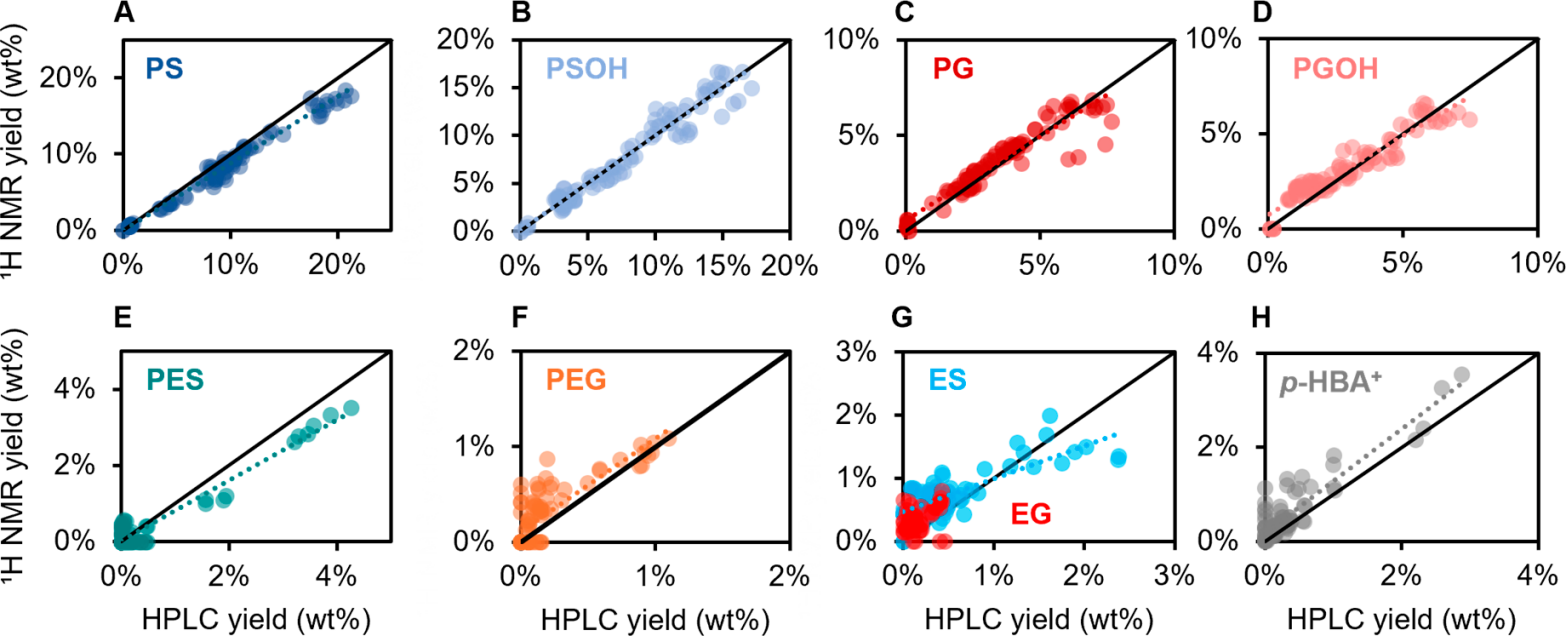
Comparison of monomer yields from ^1^H NMR and UHPLC. (A) PS (S_2/6_). (B) PSOH, (S_2/6_). (C) PG (G_2_) with correction for PEG content. (D) PGOH (G_2_). (E) PES, (S_2/6_). (F) PEG (G_2_). (G) ES, (S_2/6_), and EG calculated from the ES content and the S/G ratio of the 4-propyl products. (H) Sum of the *p*-hydroxybenzoic acid, (H_2/6_), methyl paraben (H_2/6_), and phenol (H_3/5_). RCF conditions were as previously stated with varying temperature, H_2_ pressure, reaction times, and solvent compositions. NMR specifications were as previously stated. Each data point is a single measurement of 1 RCF reaction.

### Uncommon Cases of Selectivity, and Method Limitations

The above work illustrated that the ^1^H NMR accurately measured monomer yields for RCF oils with high selectivity toward PS, PSOH, PG, and PGOH. To further investigate the limits of the method, we examined the RCF of poplar without exogenous hydrogen (i.e., “H_2_-free RCF”) which is known to strongly modulate monomer side-chain selectivity depending on the catalyst used.^9,10,18,22,23^ A high *p*-hydroxybenzoate content poplar was used to test the overlap of the phenol derivative product resonances with the G-region. When Ru/C was used under H_2_-free conditions, large peaks corresponding to PES and PEG were clearly visible in the ^1^H NMR spectrum, in line with the selectivity measured by UHPLC (Figure S27). No other signals in the ^1^H NMR spectrum could be confidently attributed to other lignin-derived monomers; only small amounts of PS and PG were measured via UHPLC (∼0.2 wt %).

When H_2_-free RCF was conducted with Pd/C, intermediate selectivity to the three saturated side chains was obtained. The S-type region displayed three singlet resonances which were integrated separately (Figure S28). Quantifications of the S-type monomers were representative of those from UHPLC. Inspection of the aromatic and aliphatic regions showed no evidence of PES or PEG, which were also not detected in UHPLC (Figure S29). Quantification of the G-type monomers proved to be more difficult. Based on the model compound spectra, it was expected that the resonances of EG and PGOH would overlap; however, the presence of phenol and perhaps other components in the RCF oil further complicated this region of the spectrum, precluding its use for quantification. In this case, the aliphatic region was integrated to determine the G-type integrals (Figure S28). These results confirm that the reliability of G monomer quantification depends strongly on reaction selectivity, with phenol-containing oils representing particularly difficult cases.

## Conclusion

A method utilizing a simple ^1^H NMR experiment was developed to rapidly quantify individual monomers from RCF oil. S-type monomers, PEG, *p*-hydroxybenzoic derivatives, and hydroxycinnamate-derived products were reliably quantified in RCF oils regardless of selectivity. The reliability of G-type monomer quantification depended on the reaction selectivity. The method adequately quantified 4-propylgauaicol and PGOH for oils with high selectivity to these products. Peak overlap between EG and PGOH made identification and quantification of these monomers difficult in cases in which these two monomers were coproduced with similar selectivity. Phenol resonances also interfered with this region; however, these two situations are expected to be uncommon for most RCF oils. The method could be applied to oils without performing liquid–liquid extraction. Combined with our previously described method for quantifying extracted lignin with the same ^1^H NMR experiment, ^1^H NMR provides a way to rapidly quantify monomers and oil without the time-consuming workup (filtration, evaporation, liquid–liquid extraction) that is typically required for RCF reactions. Ultimately ^1^H NMR provides a practical alternative or complementary method for quantification of aromatic monomers from RCF.

## Materials and Methods

A full description of the materials used and methods developed in this work are available in the SI.

## Supplementary Material



## Data Availability

Model compound spectra and examples of raw and processed NMR data are available at the Zenodo Repository: https://doi.org/10.5281/zenodo.18364528.

## Notes

Description of protocols and development of the method, ^1^H NMR spectra for model compounds, ^1^H NMR spectra of RCF oils, additional quantifications (PDF)
