# Supplementary material for: 1H NMR Quantification of Aromatic Monomers from Reductive Catalytic Fractionation
Source: ACS Sustain Chem Eng. 2026 Feb 16;14(8):3778–83. doi: 10.1021/acssuschemeng.5c10351 (PMC12958341; doi:10.1021/acssuschemeng.5c10351)
Supplement: Supplementary file 1 [file sc5c10351_si_001.pdf]

Supporting Information

^{1}H NMR quantification of aromatic monomers from reductive catalytic fractionation

Jacob K. Kenny,[1,2] Sierra Schlussel,[1] Alexander F. Benson,[1] Sean P. Woodworth,[1] Hannah M. Alt,[1] Yuriy Román-Leshkov,[3] Gregg T. Beckham[1,2,]*

1. Renewable Resources and Enabling Sciences Center, National Laboratory of the Rockies, 15013 Denver W Pkwy, Golden, CO 80401, USA

2. Center for Bioenergy Innovation, Oak Ridge National Laboratory, Oak Ridge, TN, USA

3. Department of Chemical Engineering, Massachusetts Institute of Technology, 25 Ames Street, Cambridge, MA 02139 USA

Number of pages: 44

Number of tables: 4

Number of figures: 37

A note on data presented in this work: some of NMR data presented in this work is from our previous study.[1] These include the lignin model compound spectra and the NMR spectrum of the RCF oil with high selectivity to 4-propenyl products in **Figure S27**.

Supporting methods and information

Materials

Isoeugenol, 4-propylguaiacol, 4-ethylguaiacol, phenol, *p*-hydroxybenzoic acid, methyl paraben, phenol, 1,3,5-tri-*tert*-butylbenzene, terephthalaldehyde, dimethyl terephthalate, methanol, methanol-d_4, dimethylsulfoxide-d_6, chloroform-*d*, ruthenium supported on carbon (Ru/C, 5 wt% Ru), palladium supported on carbon (Pd/C, 5 wt% Pd), platinum supported on carbon Pt/C (5 wt% Pt) and ethyl acetate were purchased from Sigma Aldrich. Acetone-d_6 was purchased from Cambridge Isotope Laboratories. 4-(3-Hydroxypropyl)-guaiacol was purchased from TCI America. 4-Propenylsyringol was purchased from AKos GmbH. 4-Ethylsyringol was purchased from AAblocks. 4-Propylsyringol and 4-(3-hydroxypropyl)-syringol were synthesized in-house according to previously described methods.[2]

Batch RCF experiments

RCF was conducted in a manner similar to our previous reports.[3] In a typical experiment and unless otherwise noted, 2 g of biomass substrate was loaded into a 75 mL Parr reactor along with 400 mg of catalyst (usually Ru/C with 5 wt% metal loading). The catalyst was wetted with 400 mg of water to prevent ignition of the organic solvent. Methanol (30 mL) was then added, and the reactor was sealed, pressure tested at 80 bar with He, and then charged with 30 bar H_2 (for experiments which used H_2). The reactor was heated for the specified reaction time; heating from room temperature to 225 °C took ~ 30 minutes. After the reaction, the reactor was cooled in an ice bath until the reaction contents were at room temperature. The reactor headspace was vented and opened and the bottom was massed to determine the post reaction mass of the reaction liquid. The reaction contents were then filtered. If LLE was not performed, the reaction mixture was syringe filtered through a 0.2 µm syringe filter; only part of the liquid content was recovered (non-quantitative transfer). Yields were calculated by measuring concentration of this solution via NMR or UHPLC and using the reaction liquid volume calculated from the pre and post reaction reactor bottom mass to calculate the total monomer amounts. When LLE was performed, the reaction mixture was first quantitatively transferred through a 2 µm filter to separate the larger biomass fragments, followed by syringe filtering with the 0.2 µm syringe filter. The reactor and filtered solids were thoroughly washed with methanol to ensure full transfer of the reactor contents. The combined solvent was evaporated in a rotary evaporator (35 °C, 80 mbar) and a 3-fold liquid-liquid extraction (LLE) was performed with ethyl acetate. The organic layers were combined and the ethyl acetate was evaporated to yield the extracted RCF oil which was dissolved in 10 mL of methanol.

Monomer yield was calculated according to equation (1) based on the total lignin content in the biomass initially loaded:

$$y_i = \frac{C_i * V}{m_{biomass} * X_{lignin}} \tag{1}$$

where:

- y_i is the yield of an individual lignin monomer
- C_i = concentration of the lignin monomer measured by the analytical method (e.g. UHPLC or ^{1}H NMR),
- V = the total volume of RCF oil sample (typically 10 mL)
- $m_{biomass}$ = mass of biomass loaded in the RCF reactor initially (~ 2 g),
- X_{lignin} = mass percent of lignin in the biomass measured by compositional analysis.

The total monomer yield is then expressed as the sum of the individual monomer yields.

$$Y_t = \sum y_i \tag{2}$$

Measurement of lignin monomers with ultra-high pressure liquid chromatography (UHPLC)

All poplar and pine reactions were analyzed with UHPLC according to the published method on the protocols.io.[4] Example chromatograms are shown in **Figure S30**.

Measurement of lignin monomers with gas chromatography with flame ionization detection (GC-FID)

Reactions on switchgrass were analyzed using the published method for monomer analysis using GC-FID on protocols.io.[5]

Preparation of a sample for NMR

To prepare an RCF sample for NMR, an aliquot (0.1-1 mL) of the filtered reaction liquor or extracted RCF oil solution was added to a 4 mL vial. The solvent was evaporated under flowing N_2 for ~ 5 min or until no solvent could be seen in the vial. Note that we used air in our previous report; both air or N_2 are expected to be acceptable for the purpose of evaporating the solvent .[1] Then, 0.5-1 mL of acetone-d_6 containing an internal standard (usually TTB) was added to the vial and the vial was shaken to redissolve the oil. In cases in which the RCF oil was not fully soluble, typically because an LLE was not performed leading to a higher content of water-soluble compounds, 0.1 mL of methanol-d_4 was added to fully solubilize the oil. The sample was then transferred to an NMR tube. In general, it is also recommended that a small amount of methanol-d_4 (~ 0.1 mL) be added to the NMR sample to reduce the intensity of the phenolic-OH resonances in the aromatic region, especially near the TTB internal standard.

General NMR information

NMR spectra were acquired primarily on a Bruker Avance 400 MHz spectrometer equipped with a Prodigy Cryoprobe, although some of the model compound experiments were acquired on a Bruker Avance III 600 MHz spectrometer using a room-temperature broadband probe or a Bruker Avance AV4 NEO Nanobay NMR spectrometer equipped with a standard 5 mm room-temperature BBFO probe. TopSpin 3.6 and IconNMR automation software were used for data acquisition. Model compound NMR experiments were typically acquired on solutions in the range of 1-10 mg/mL. ^{1}H NMR spectra were acquired using the zg30 pulse sequence with, 32 scans, a recycle delay (d_1) of 3 s, a sweep width of 13 ppm, 2 dummy scans, TD (size of FID) = 64k, acquisition time of 6.30 s, and an O1P = 6.175. Quantification is typically sensitive to the d_1 parameter, and this was investigated for a range of internal standards, 3 was found to be sufficient for TTB (*vide-infra*).

NMR spectra were processed using exponential apodization with a line broadening parameter of 0.3 Hz. Zero filling was performed to 1 level (zero filled to 2x initial spectrum size). Automatic baseline correction was performed using Nnova's multipoint baseline feature.

Resonances were typically integrated using the "Sum" mode in Mnova. However, when peak deconvolution was desired, the "Peaks" mode was selected instead (*vide infra*).

The following equation (3) was used to calculate the concentration of the lignin analyte from the integrals.

$$C_i = MW_i * \left(\frac{V_s}{V_A}\right) * C_{IS} * \left(\frac{I_i}{N_i}\right)\left(\frac{N_{IS}}{I_{IS}}\right) \quad (3)$$

where:

- MW_i = the molecular weight of the lignin analyte
- V_S = volume of the NMR sample (0.5-2 mL)
- V_A = the volume of the RCF sample solution aliquot which was deposited and evaporated (~ 0.2 mL)
- C_{IS} = concentration of the internal standard in the deuterated solvent mixture (~ 1 mg/mL)
- I_i = integral of the lignin analyte protons
- I_{IS} = integral of the internal standard protons
- N_i = number of protons giving rise to the integrated lignin analyte aromatic resonance (2 for syringyl-type monomers, 1 for guaiacyl-type monomers)
- N_{IS} = number of protons giving rise to the integrated internal standard aromatic resonance (3 for TTB)

Example:

An RCF reaction was conducted on 2 g of poplar ($m_{biomass}$) with a lignin content of 23.8 wt% (X_{lignin}). After executing the RCF method described above, the filtered/extracted RCF oil was dissolved in 10.2 mL of methanol (V). An aliquot with the volume 0.5 mL (V_A) of this solution was evaporated in a vial. A solution of TTB was prepared with 21.4 mg of TTB and 18.22 g of acetone-d_6, leading to a concentration (C_{IS}) of 4.02 μmol/mL after accounting for the purity of TTB (97%). 1 mL of this internal standard solution was added to the evaporated RCF oil sample vial to dissolve the RCF oil, and the sample was transferred to an NMR tube. A ^{1}H NMR experiment was conducted and the 2/6 resonance of 4-propylsyinrgol was integrated to give a value of 1.73 relative to the internal standard of 1. The concentration of 4-propylsyringol was calculated as:

$$C_{PS} = 196.2\frac{\text{mg}}{\text{mmol}} * \left(\frac{1\text{ mL}}{0.5\text{ mL}}\right) * \left(0.00402\frac{\text{mmol}}{\text{mL}}\right) * \left(\frac{1.73}{2}\right)\left(\frac{3}{1}\right) = 4.10\text{ mg/mL}$$

The yield of 4-propylsyringol was then calculated as

$$y_i = \frac{4.10\frac{mg}{mL} * 10.2\ mL}{2000\ mg * 0.238} = 8.8\ wt\%$$

Development of the NMR method

To utilize NMR as a quantification method, several experimental considerations must be addressed. Below are descriptions of the development process of the NMR method and recommendations for these key parameters, although some flexibility is available to the user, such as with sample concentration.

Choice of internal standard: The choice of internal standard is extremely important to ensure proper quantification. An internal standard should be inert, soluble in the NMR solvent, free from interference/overlap with other resonances, and ideally nonvolatile to ensure the concentration does not change during sample preparation and measurement. In our previous report, we used 1,3,5-tri-*tert*-butylbenzene (TTB) as an internal standard.[1] TTB was chosen because it was soluble in the NMR solvent (acetone-d_6) and its aromatic peak was sufficiently well resolved from the resonance of the RCF oil. An additional constraint in that work was that it was used as the internal standard on GC-FID, which required it be detectable but not overlap with lignin-derived monomers. Although it led to sufficient quantification, TTB had several drawbacks that motivated exploration of alternatives:

1. Its resonance position (~7.28 ppm in acetone-d_6) was close, and perhaps occasionally overlapping with broad sample resonances. These were later determined to be due to the guaiacyl phenolic-OH groups, which could be abated by added a small amount of D_2O or methanol-d_4
2. The purity of the commercially available sample was low (minimum 97%) and contained an impurity that was insoluble in many organic solvents.
3. TTB is more expensive than many other organics that could possibly serve as standards ($363 US for 10 g from Sigma-Aldrich at the time of writing).

Given these reasons, several other compounds were investigated to potentially serve as internal standards, and the comparison of their ^{1}H NMR spectra with that of RCF oil is shown in **Figure S31**.

Terephthalaldehyde is non-toxic and displays distinguishable resonances at 8.147 (4H, s, aromatic) and 10.192 (2H, s, aldehyde) ppm. It is also cheaper than TTB ($110 for 500 g from Sigma Aldrich at the time of writing) and available in high purity (99%). Terephthalaldehyde was used for quantification of RCF oils generated using Pd/C under H_2-free conditions. The major downside of terephthalaldehyde was the long T_1 relaxation of both the aromatic and aldehyde protons (8-10 s), requiring correspondingly long d_1 times (and therefore longer experiment times) in the ^{1}H NMR quantification experiments.

Dimethyl terephthalate shares properties similar to terephthalaldehyde as it is fairly non-toxic, cheap ($31 for 500 g from Sigma Aldrich at the time of writing), and available in high purity (99%). It displays two resonances at 3.926 (6H, s, methoxy) and 8.119 (4H, s, aromatic) ppm. The aromatic resonance would be preferred over the methoxy resonance since the methoxy peak would overlap extensively with signal from lignin-derived compounds in that chemical shift range. Although not used for quantification in this work, dimethyl terephthalate could likely be a preferred internal standard, however dedicated NMR experiments should be performed to determine the required d_1 time to ensure quantification. Dimethyl terephthalate was previously chosen as a preferred internal standard.[6]

Several other compounds were also tested for their chemical shifts but showed some undesirable aspects. 1,3,5-tribromobenzene displays a resonance at 7.805 ppm (3H, s, aromatic) which may overlap with *para*-hydroxybenzoic acid or methyl paraben. 1,3,5-Trimethoxybenzene displays a single resonance at 6.085 ppm (3H, s, aromatic) but this may overlap the α/β peaks of unsaturated compounds.

NMR solvent: The NMR solvent is an important component of the method. The selected solvent must fully dissolve the NMR sample and the residual solvent peaks should not interfere with the sample resonances of interest. It can also affect the chemical shifts of the sample observed in the NMR experiment. Previously, we had used acetone-d_6 as the solvent as it is cheap ($248 per 50 g at the time of writing) and has a clear residual solvent peak that does not interfere with lignin analysis. We occasionally encountered samples which were not fully soluble in acetone, in which cases methanol-d_4 was added. Although pure methanol-d_4 is also a suitable solvent, it is 2-3x more expensive than acetone-d_6. Chloroform-d is perhaps the most common NMR solvent, however the residual solvent peak (~7.24 ppm) falls near those arising from the RCF oil. It also may not be guaranteed to fully dissolve the lignin-derived compounds given its lack of polarity. Dimethyl sulfoxide is also cheap, but the high viscosity can leads to peak broadening in the NMR spectrum and the high boiling point makes recovery of the oil from the NMR sample if desired

(not pursued here) difficult.[7] Solvent selection may alter the exact peak position and the relaxation behavior; however it appears that acetone-d_6 is a still the best choice. The NMR spectra of RCF dissolved in these different NMR solvents is shown in **Figure S32**.

Relaxation parameters: Quantitative NMR requires proper selection of the recycle delay (d_1) in order to make accurate peak integrations. This parameter depends on the relaxation characteristics of the sample which can be characterized by the spin-lattice relaxation time constant (T_1). The rule of thumb is to set the d_1 parameter to 5x the longest T_1 for a zg90 experiments, although a shorter T_1 can be used for experiments with a smaller flip angle such the zg30 experiment, in which case the d_1 can be set to 3x T_1.

To ensure proper quantification, the T_1 values of the internal standards and various peaks in the ^1H NMR spectrum of RCF oils were measured through an inversion recovery experiment using both 1,3,5-tri-*tert*-butylbenzene and terephthalaldehyde as internal standards. As an additional check, ^1H NMR experiments (standard zg30 pulse sequence) were also run with varying d_1 values (**Figure S35**). Lignin peaks were found to have T_1 values between 3-4 s, however the internal standards TTB and TPA had longer T_1 values. The T_1 for the aromatic protons on TTB (which were used for quantification) was 4.81 s. However, acquiring experiments with varying delays (d_1) showed only slight change in the integrals for d_1 values longer than 3 s, and thus the spectra obtained throughout this work used a d_1 value of 3 s, although a longer d_1 of ~15 s may be preferable for RCF oils of unknown composition. The T_1 relaxation time constants of the aldehyde and aromatic protons on TPA were measured to be 10.22 and 8.31 s, respectively. Performing similar experiments to with longer d_1 values using TPA showed a larger dependence on d_1, and therefore a longer delay (30 s) is necessary for quantitative NMR. When TPA was used for quantification, d_1 was set to 30 s.

Sample concentration: A primary advantage of NMR is that quantification can be obtained from small amounts of sample. However, in typical experiments where RCF is performed using 2 g biomass, sample availability may not be a constraining factor. In general, as sample concentration increases, signal/noise can increase, making detection of low abundance possible. However, RCF monomer resonances are surrounding by other lignin resonances, and increasing concentration does not increase the prominence of the monomer peaks relative to the surround signal. Thus, we sought to explore the optimal sample concentration. Samples were typically prepared with an RCF oil concentration in the NMR tube of ~ 10 mg/mL. The data in **Figure 3** were measured with concentrations ranging from 5-20 mg/mL, however the method was previously applied to samples up to ~ 40 mg/mL without any noticeable change in the NMR spectra.[1]

One important factor is that samples prepared from lower amounts of liquid transferred for evaporation were apparently more susceptible to over-drying. Samples were prepared by adding varying amounts of extracted RCF oil in methanol (0.1, 0.2, 0.3, 0.5 mL) to 4 mL vials. The vials were then dried under N_2 for the same amount of time until the sample with 0.5 mL was dry. Upon NMR analysis, the G_2 peak for 4-propylguaiacol showed an inverse trend with the amount of sample added to the vial, with the 0.1 mL sample showing the lowest integrations and the 0.5 mL sample showing the highest (**Figure S34**). The samples were dried for approximately 15 minutes. The good agreement of 4-propylguaiacol yields between NMR and UHPLC (**Figure 3C**) combined with the observation that other solutions were prepared with varying amounts of sample and did not show this trend suggest that this is not a widespread issue. This reinforces that drying time is an important parameter and care should be taken to only subject the sample to drying until the solvent has been removed.

Peak identifications and integration ranges: In initial experiments, large, broad signals were often observed in the spectra of RCF oils in the ranges of 6.86-6.92 and 7.22-7.26 ppm. These were eventually shown to be the syringyl and guaiacyl phenolic hydroxyl groups, respectively (see model compound spectra in **Figure S1-S20**). All lignin model compounds tested showed an additional downfield resonance aside from the assigned aromatic peaks; the phenolic hydroxyl group resonances for the unsaturated monomers 4-propenylsyringol (δ of phenolic hydroxyl group: 7.13 ppm) and isoeugenol (δ of phenolic hydroxyl group: 7.49 ppm) were shifted downfield compared to the saturated model compounds. In the RCF oils, the OH character of these resonances was confirmed by addition of D_2O or methanol-d_4, which eliminated the resonances (**Figure S33**).

All peak identification was performed by comparing the spectra of RCF oils and model compounds, in addition to examining the spectra of oils with known selectivity. Integration was performing using the "sum" mode in Mnova, rather than a deconvolution algorithm. Examples of integration ranges are shown in **Figure S36**. Peaks could not be integrated with wide regions due to the location of other signals. Therefore, integration regions were determined based on the judgment of the user and agreement with UHPLC quantifications for select spectra before being applied to additional oils as shown in **Figure 3**. Integration ranges were changed depending on selectivity of the reaction since larger peaks required wider integration ranges to fully capture the integral.

The syringyl integrals are typically identifiable and can be integrated regardless of the reaction selectivity. In the case of intermediate selectivity, such as the co-production of 4-ethylguaiacol and 4-(3-hydroxypropyl)-guaiacol, or when additional resonances complicate the aromatic region, an alternative strategy may be needed for quantification of the guaiacyl monomers. Such a case was presented in the main text for reactions with Pd/C with and without 30 bar H_2. In these cases, we evaluated alternatives to the direct integration of the aromatic resonances. For 4-propylguaiacol, information can be gained from either the γ (CH_3) or β (CH_2) protons. Simple subtraction of the area of 4-propylsyringol area from either γ or β aliphatic integrals resulted in

an over quantification compared to UHPLC values (**Figure S27**). Performing peak deconvolution of the γ CH_3 peak led to better agreement between the UHPLC and the NMR values. Similar results were obtained for quantifying 4-ethylgauaicol from these reactions (**Figure S37**). As mentioned in the main text, 4-propanolgauaicol aliphatic peaks overlap with other monomer resonances. However, the integral of the β protons at 1.77 ppm can be utilized by subtracting out the contributing of 4-(3-hydroxypropyl)-syringol from the area.

Recommended protocol for monomer quantification with ^1H NMR spectroscopy

The following method should be applicable to most oils. It assumes that the RCF oil is dissolved in a volatile non-deuterated solvent such as methanol. This solution should be filtered so that solid particles are not transferred into the NMR sample.

1. Add an aliquot of RCF oil into a small container in which the solvent can be evaporated and the sample can be capped after deuterated solvent addition it can be shaken to dissolve. We used 4 mL vials with caps. The volume of the aliquot should be chosen to produce an NMR sample with an RCF oil concentration in the range of 15-25 mg/mL. Aliquots used in this work were typically in the range of 0.1-0.5 mL. The volume (or mass) of the aliquot must be known so that the concentrations obtained from NMR quantifications can be multiplied by the total sample volume (or mass) to enable calculation of yields or total monomer amounts.
2. Evaporate the solvent. We performed this evaporation under flowing air or nitrogen gas at room temperature, however a rotary evaporator with a connector to enable evaporation of the specific container being used could also work. Care should be taken to not over-dry the sample which may prompt evaporation of 4-propylguaiacol.
3. Prepare a solution of internal standard such as TTB dissolved in acetone-d_6. We used 99.9% atom % D. We aimed for a concentration of 1 mg internal standard/mL; however both the mass of the internal standard and the mass of the acetone-d_6 solvent should be measured to 0.1 mg to enable accurate calculation of concentration.
4. Add the desired amount of internal standard solution to the evaporated oil. The amount of solution added must be measured/known. We used volumetric pipettes for this addition. The volume transferred was typically between 0.5-1 mL.
5. Close the container and shake to fully dissolve the NMR sample. If the sample is not fully dissolving, methanol-d_4 can be added to the sample until the sample is fully dissolved. We added in aliquots of 0.1 mL; typically, only 1 aliquot (0.1 mL) was needed. Addition of methanol-d_4 was typically needed for samples which had not been subjected to liquid-liquid extraction or for switchgrass RCF oil samples.
6. Once the sample is dissolved, transfer the NMR sample to an NMR tube. We performed this transfer using a glass pipette. Since the sample is homogeneous, the transfer does not need to be complete/quantitative; however the NMR sample volume should be adequate to satisfy the sample volume requirements of the NMR spectrometer that is being used (often minimum of 0.5 mL).
7. Perform standard locking, tuning, and shimming prior to data acquisition. Acquire a 1-D ^1H NMR scan using a standard pulse sequence such as zg30. If zg90 is used, a longer d1 will need to be selected (generally recommended to be 5x longest T1). Acquisition parameters are included in **Table S1**.
    a. The required d1 will depend on the internal standard being used. We used a d_1 of 3 s in this work when using TTB as the internal standard as no differences were observed in the spectra/quantifications when acquired at different d_1 values higher than 3 s, however it is generally recommended that a d_1 of 3x the longest T_1 in the sample is used when using a zg30 pulse sequence. A dependence on d_1 was seen when using terephthalaldehyde, in which case d_1 was set 30 s. It is recommended to first measure T_1 values of the sample before performing quantification.

Table S1. Acquisition parameters for the 1-D ^1H NMR.

Parameter	Value
Pulse sequence	zg30
Number of scans	32
Dummy scans	2
Acquisition time	6.3 s
Spectral width	13 ppm
Sample temperature	298 K
Data points (TD)	64K

8. After acquisition, process the NMR data. Mnova was used in this work to analyze the NMR data. All spectra were calibrated to the residual solvent signal; for acetone-d_6 this was located by the quintet at δ = 2.05 ppm.
9. Integrate internal standard peak.
10. Identify and integrate prominent lignin monomer peaks using **Table S2 and S3.**

a. First identify the syringyl monomers based on their singlet resonances (6.465-6.505 ppm in acetone-d_6). RCF oils will commonly exhibit large resonances for 4-propylsyringol and 4-(3-hydroxypropyl)-syringol. The resonance for 4-ethylsyringol will be located between these two peaks. These syringyl monomers can typically be integrated without consulting other parts of the spectrum for additional information. Integrate the identifiable peaks.

b. Investigate the aromatic region for unsaturated products. 4-Propenylsyringol will exhibit a singlet resonance at 6.664 ppm **(Figure S4, S27**). Isoeugenol will display a clear doublet at 6.994 ppm if present (**Figure S3**). If these resonances appear to be present, confirm that the sample contains unsaturated compounds by identifying the α and β proton resonances at 6.725-6.349 ppm and 6.054-6.151 ppm, respectively. If these resonances are also present, integrate aromatic resonances of the unsaturated monomers.

c. Quantify the guaiacyl products. If the major products can confidently be identified from the aromatic region, integrate the aromatic peaks. This is typically the case for oils rich in 4-proyplguaiacol and 4-(3-hydroxypropyl)-guaiacol. However, if overlap occurs, the aliphatic region may need to be utilized for quantification (**Figure S37**).

d. If both 4-propylguaiacol and isoeugenol are present, the 4-propylguaiacol integral should be corrected for overlap (**Figure S21**). Note that this correction has only been applied for oils which exhibit relatively low yields of isoeugenol (<1 wt%) compared to 4-propylguaiacol.

$$I_{PG-c} = I_{PG} - 0.5 * I_{IEG} \quad (4)$$

e. If the major monomers appear to be 4-ethyl and 4-(3-hydroxypropyl) substituted from the syringyl region, a large degree of overlap will likely be encountered for the resonances of 4-ethylguaiacol and 4-(3-hydroxypropyl)-guaiacol. In this case, 4-ethylguaiacol was calculated from the aliphatic β peak (1.15-1.20 ppm) using the "peaks" integration mode in MNova, which deconvolutes the combined integral of 4-ethylguaiacol and 4-ethylsyringol (**Figure S37**). The success of this method relies on this integration region being fully attributable to the β protons of the 4-ethyl monomers, and thus any overlap with resonances of similar compounds will inflate this integral resulting in over-quantification.

f. To quantify 4-propylguaiacol from the aliphatic region, a similar use of the "Peaks" method for deconvolution of the γ CH_3 protons was used (**Figure S37**).

g. The "Peaks" method could not be applied to quantify 4-(3-hydroxypropyl)-guaiacol due the lack of definition in the aliphatic peaks. To obtain a quantification, the integral of the β protons at ~1.77 ppm was used, however this led to substantial over quantification of 4-(3-hydroxypropyl)-guaiacol in the two samples in which it was attempted (**Figure S37**). Therefore, use of the aliphatic region in general should be approached with caution and potentially only used qualitatively. The corrected integral was calculated by subtracting the integral of 4-(3-hydroxypropyl)-syringol from the integral from 1.73-1.82. Since the β resonance and the 2/6 resonance of 4-(3-hydroxypropyl)-syringol both arise from 2 protons, adjustment is not needed.

$$I_{PGOH-c} = I_{1.77} - I_{PSOH} \quad (5)$$

11. For quantification of hydroxycinnamate molecules from herbaceous feedstocks such as switchgrass, refer to **Table S4**.

a. If using an alcohol solvent such as methanol, determine if any free (un-esterified) carboxylic acids are present. This is most easily done by comparing the peak locations of 3-(4-hydroxyphenyl)-propionic acid and methyl-3-(4-hydroxyphenyl)-propanoate, or 3-(4-Hydroxy-3-methoxyphenyl)-propionic acid and methyl 3-(4-hydroxy-3-methoxyphenyl)-propanoate.

b. Integrate the hydroxycinnamate peaks detailed in **Table S4**.

Table S2. Integration regions used for standard quantification of RCF oils rich in 4-propyl and 4-(3-hydroxypropyl)-substituted products corresponding to **Figure 1**. NMR solvent was 1:10 methanol-d_4/acetone-d_6.

	Range	# H
Methyl paraben	7.898 .. 7.868	2
TTB	7.295 .. 7.269	3
Phenol	7.203 .. 7.155	2
G region	7.200 .. 6.600	3
Isoeugenol	6.994 .. 6.986	1
4-(3-Hydroxypropyl)-guaiacol	6.814 .. 6.802	1
4-Propylguaiacol	6.790 .. 6.778	1
4-Propenylsyringol	6.665 .. 6.660	2
S-region	6.600 .. 6.200	2
4-(3-Hydroxypropyl)-syringol	6.500 .. 6.492	2
4-ethylsyringol	6.492 .. 6.487	2
4-propylsyringol	6.479 .. 6.466	2
4-propyl α & 4-(3-hydroxypropyl) α	2.614 .. 2.452	2
4-propenyl γ/4-(3-hydroxypropyl) β	1.819 .. 1.733	3/2
4-propyl β	1.630 .. 1.552	2
4-ethyl β	1.198 .. 1.147	3
4-propyl γ	0.932 .. 0.875	3

Table S3. Integration regions used for standard quantification of RCF oils rich in 4-propyl and 4-(3-hydroxypropyl)-substituted products corresponding to **Figure 2**. NMR solvent was acetone-d_6. Phenol was not observed in the ^{1}H NMR spectra.

	Range	# H
Methyl paraben	7.939 .. 7.851	2
TTB	7.291 .. 7.277	3
G region	7.200 .. 6.600	3
Isoeugenol	6.994 .. 6.984	1
4-(3-Hydroxypropyl)-guaiacol	6.814 .. 6.802	1
4-Propylguaiacol	6.791 .. 6.779	1
4-Propenylsyringol	6.666 .. 6.660	2
S region	6.600 .. 6.200	2
4-(3-Hydroxypropyl)-syringol	6.500 .. 6.493	2
4-Ethylsyringol	6.490 .. 6.486	2
4-Propylsyringol	6.479 .. 6.469	2
4-Propyl α & 4-(3-hydroxypropyl) α	2.616 .. 2.448	2; 2
4-Propenyl γ & 4-(3-hydroxypropyl) β	1.822 .. 1.745	3; 2
4-Propyl β	1.646 .. 1.533	3
4-Propyl γ	0.937 .. 0.873	3

Table S4. Integration regions used for quantification of coumarates and ferulate compounds in RCF reactions using switchgrass as the substrate.

	Range	# H
Coumaric acid/methyl coumarate	7.577 .. 7.515	2
Ferulic acid/methylferulate	7.368 .. 7.304	1
3-(4-Hydroxyphenyl)-propionic acid	7.105 .. 7.037	2
Methyl-3-(4-hydroxyphenyl)-propanoate	7.089 .. 7.002	2
3-(4-Hydroxy-3-methoxyphenyl)-propionic acid*	6.889 .. 6.852	1
Methyl 3-(4-hydroxy-3-methoxyphenyl)-propanoate	6.863 .. 6.824	1
4-Ethylphenol	7.053 .. 6.963	2

*This integral overlaps with other guaiacyl monomers and would likely be difficult to assign. Here, RCF was conducted in methanol and therefore the hydroxycinnamates were fully esterified.

Supporting figures

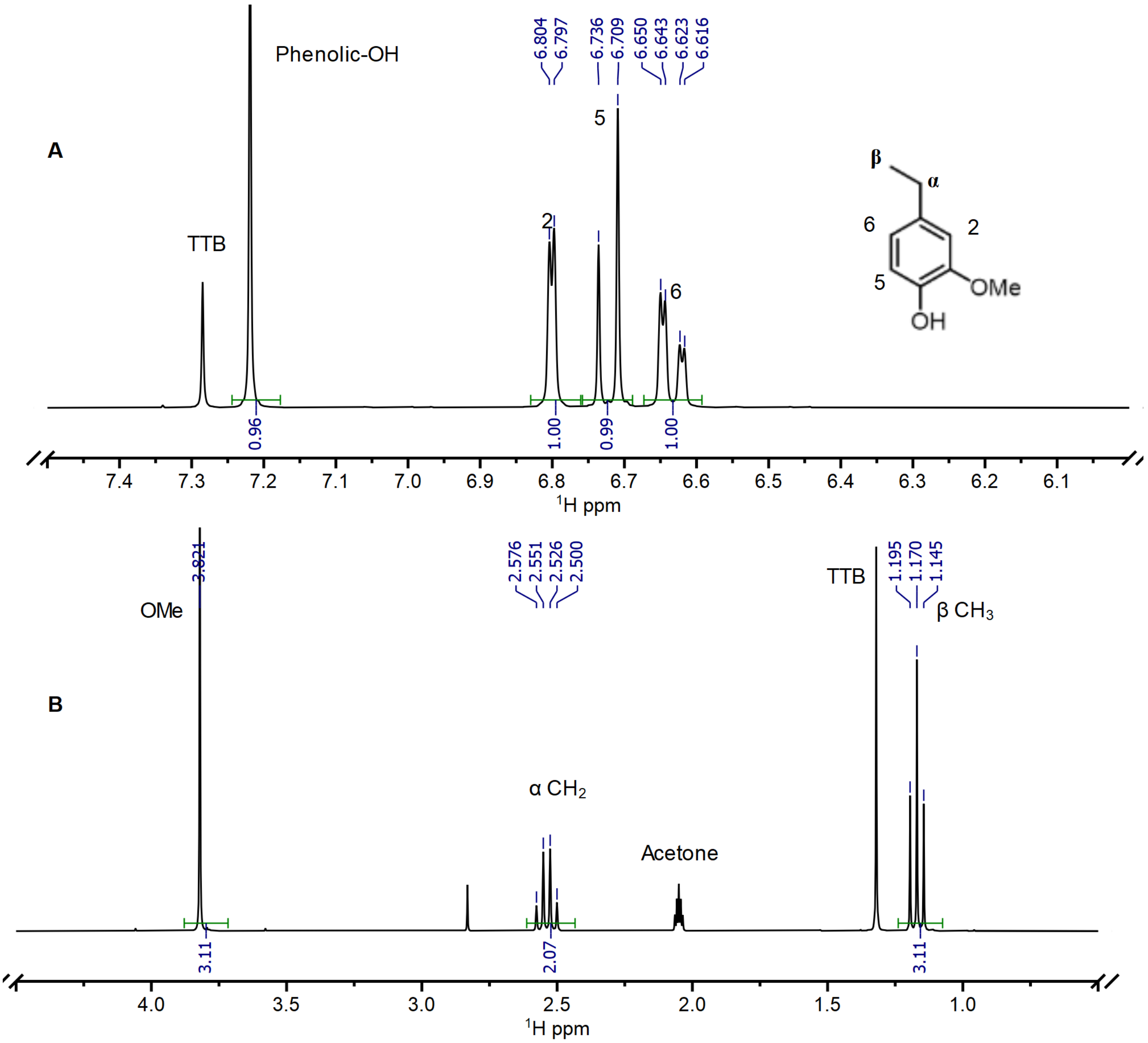

Figure S1. ^{1}H NMR spectrum of 4-ethylguaiacol (EG). (**A**) aromatic region. (**B**) Aliphatic region. ^{1}H NMR, acetone-d_6, 300 MHz.

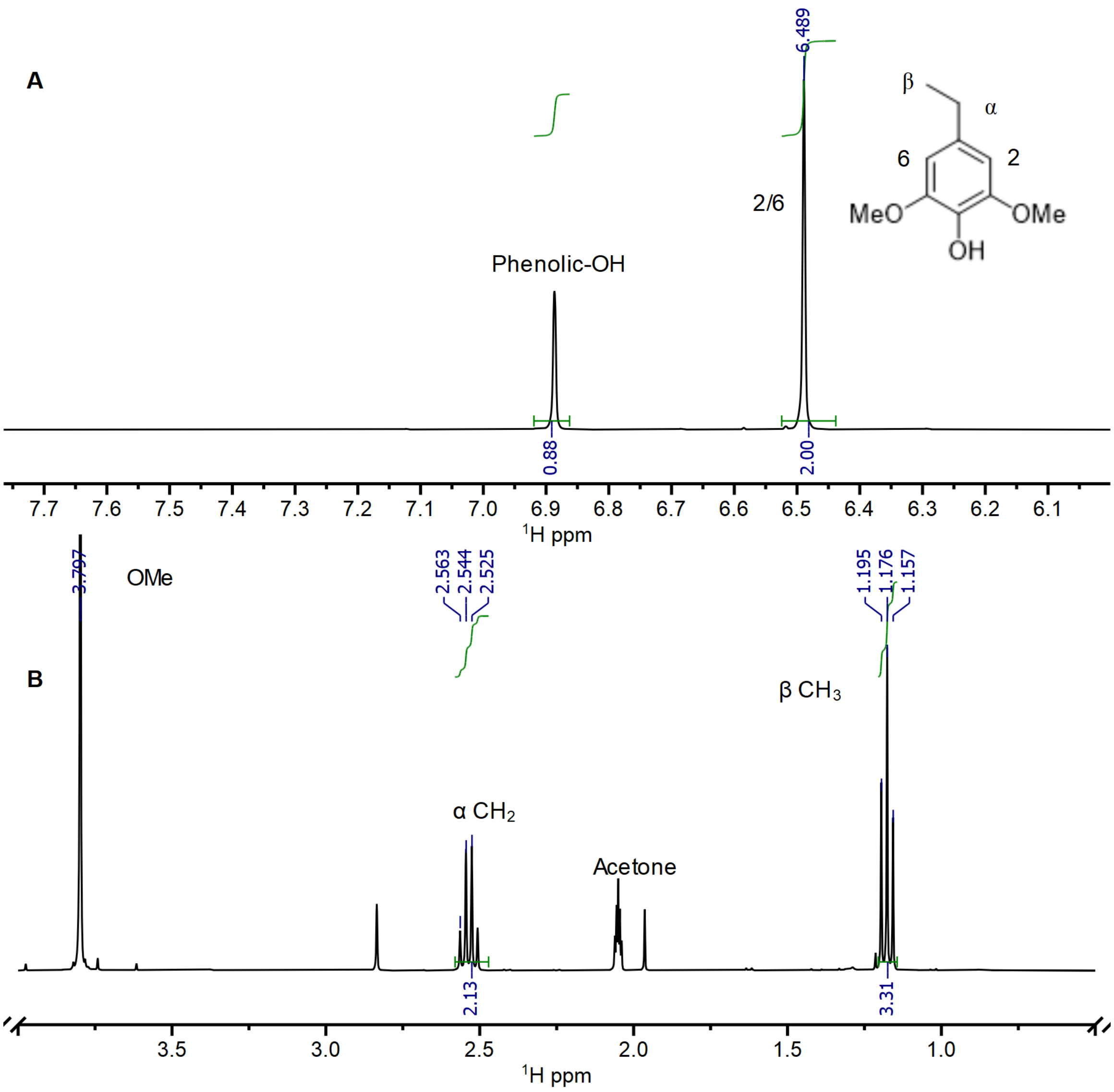

Figure S2. ¹H NMR spectrum of 4-ethylsyringol (ES). (**A**) aromatic region. (**B**) Aliphatic region. ¹H NMR, acetone-d_6, 400 MHz.

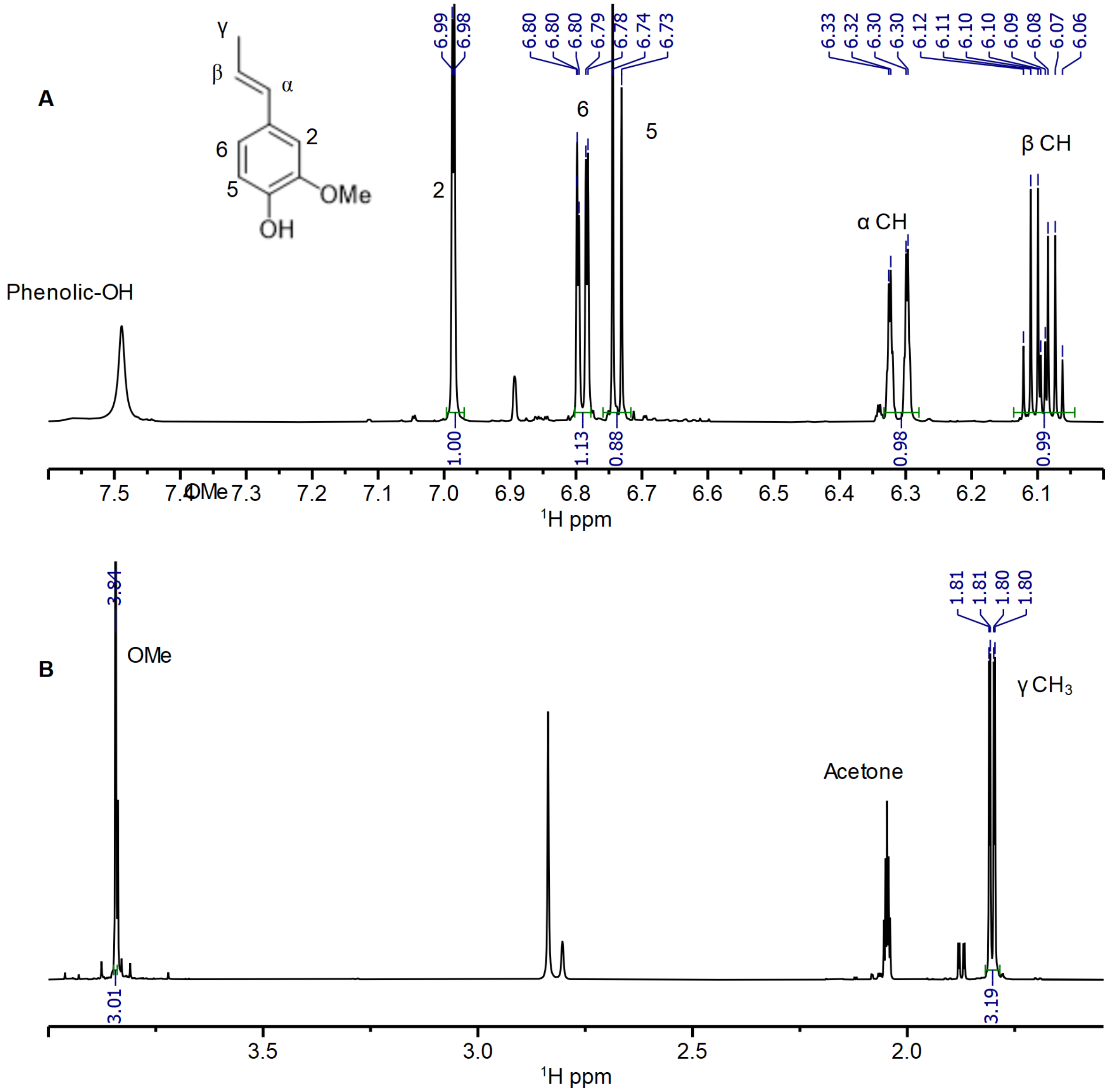

Figure S3. ^{1}H NMR spectrum of isoeugenol (PEG). (**A**) aromatic region. (**B**) Aliphatic region. ^{1}H NMR, acetone-d_6, 600 MHz.

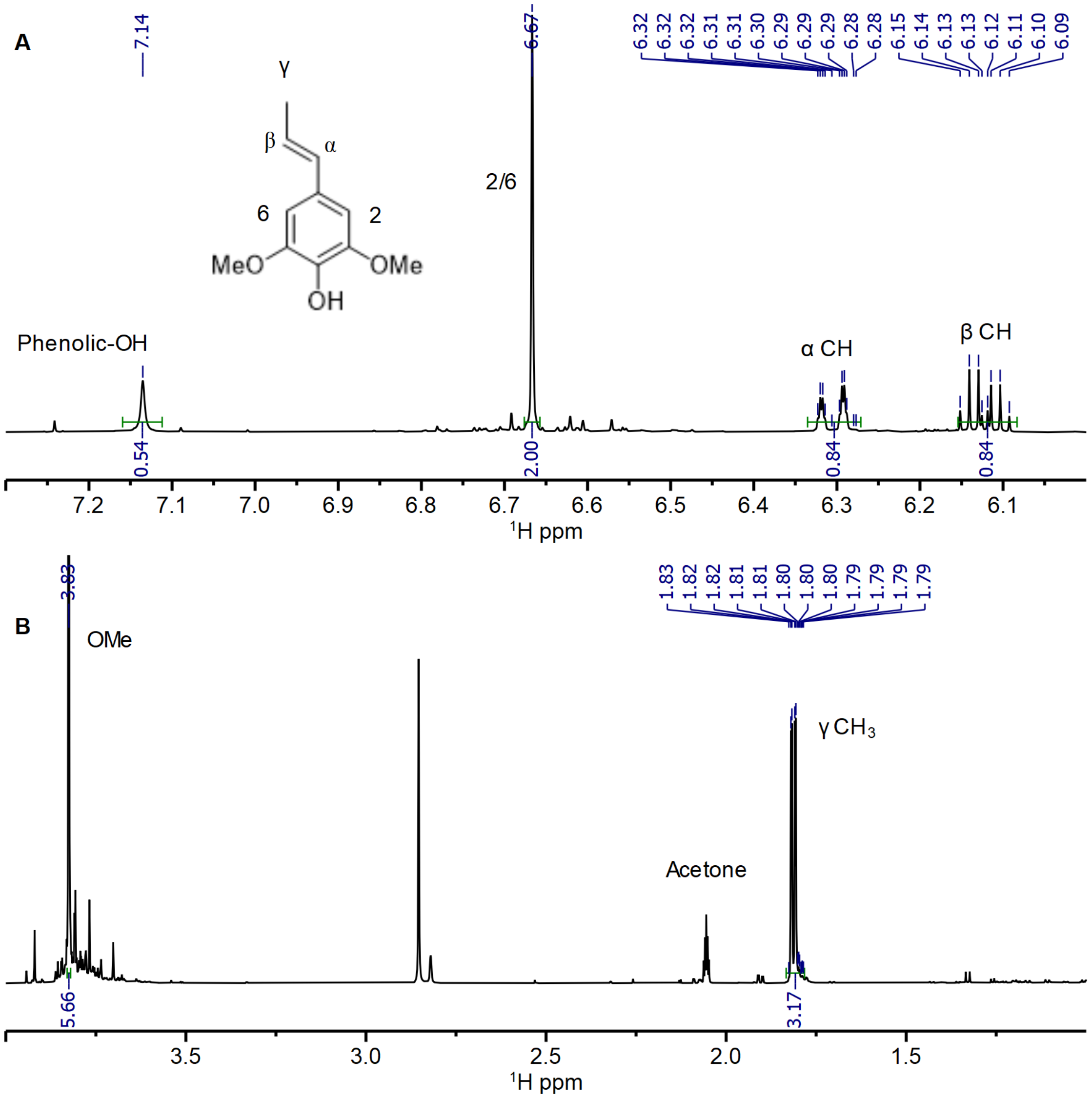

Figure S4. ^{1}H NMR spectrum of 4-propenylsyringol (PEG). (**A**) aromatic region. (**B**) Aliphatic region. ^{1}H NMR, acetone-d_6, 600 MHz. The additional resonances in the aromatic region and around the methoxy peak indicate that the compound was not pure, likely due to degradation. In this case, the "Peaks" method was used for integration. The spectrum was used as a reference for the positions for the 4-propenylsyringol peaks in RCF oil.

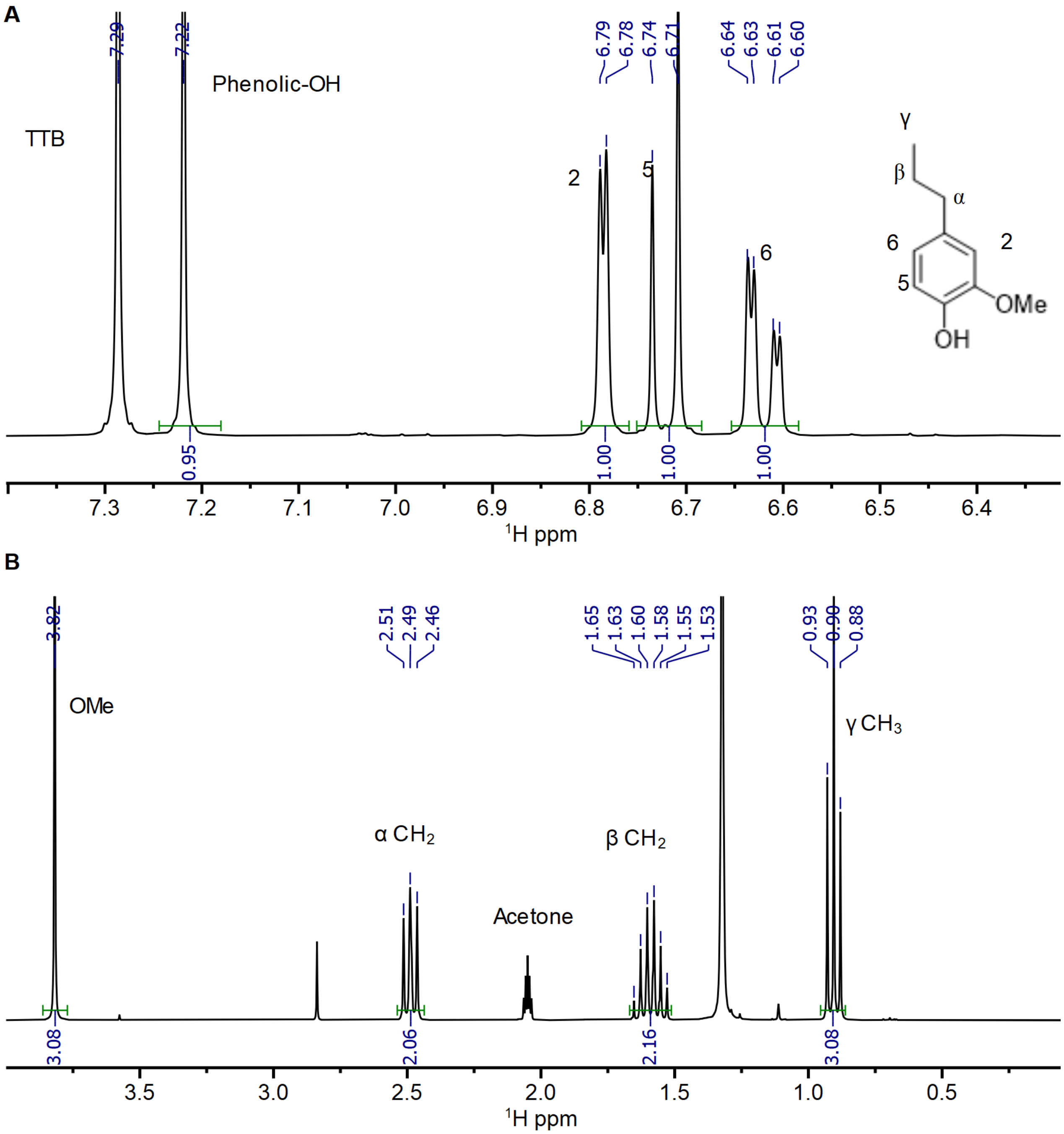

Figure S5. ¹H NMR spectrum of 4-propylguaiacol (PG). (**A**) aromatic region. (**B**) Aliphatic region. ¹H NMR, acetone-d_6, 300 MHz.

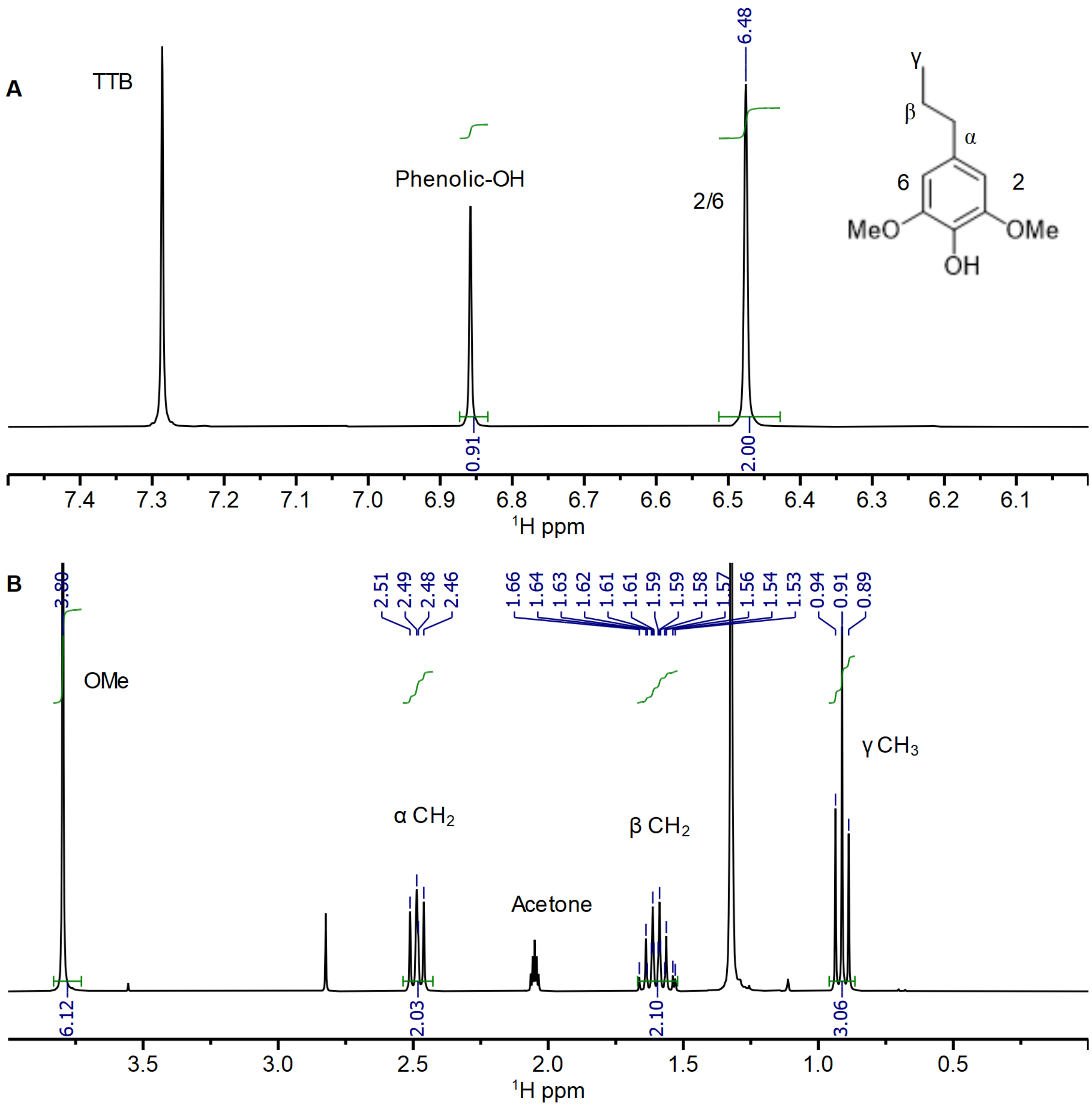

Figure S6. ^{1}H NMR spectrum of 4-propylsyringol (PS). (**A**) aromatic region. (**B**) Aliphatic region. ^{1}H NMR, acetone-d_6, 300 MHz.

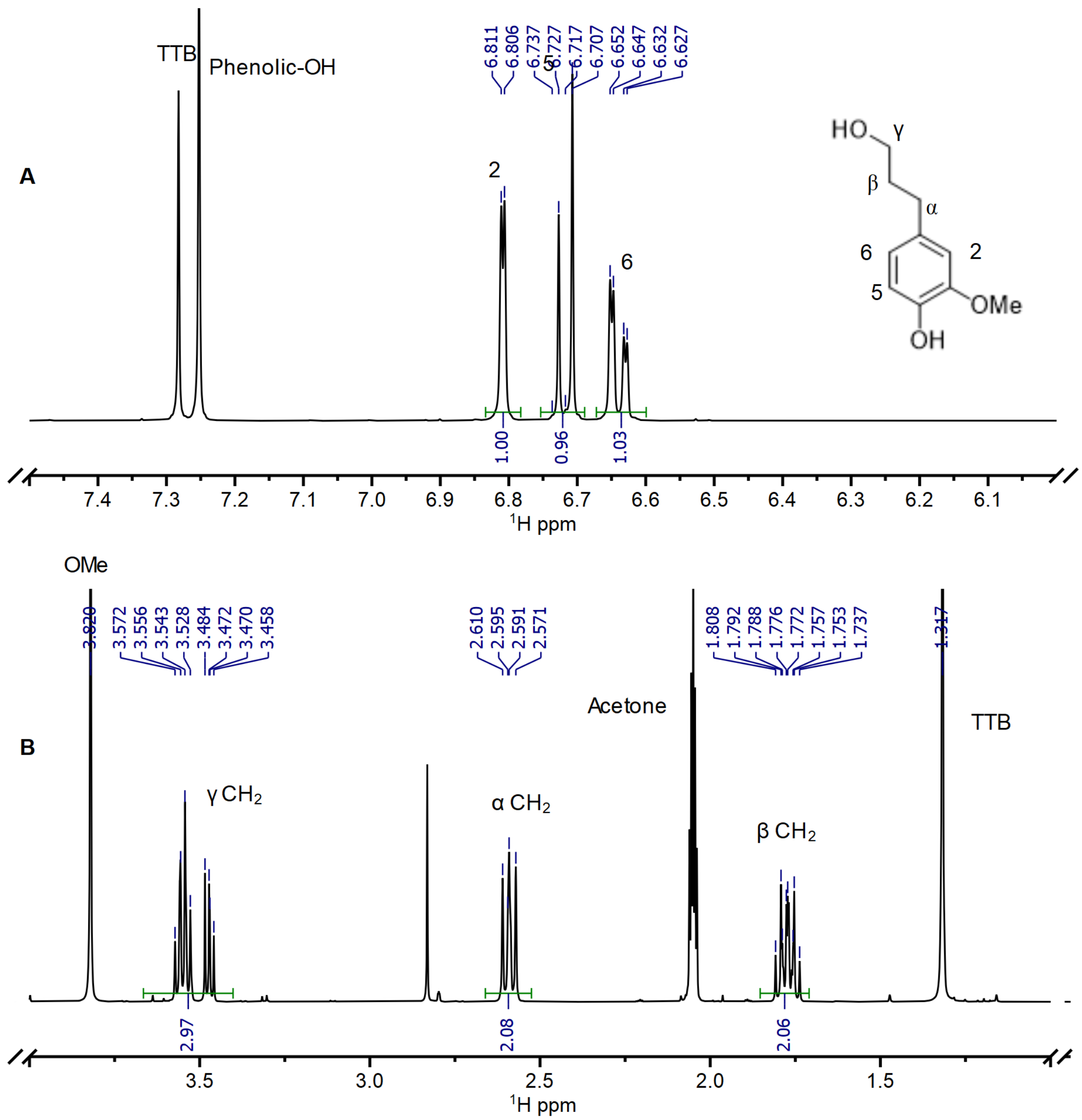

Figure S7. ^{1}H NMR spectrum of 4-(3-hydroxypropyl)-guaiacol (PGOH). (**A**) aromatic region. (**B**) Aliphatic region. ^{1}H NMR, acetone-d_6, 400 MHz.

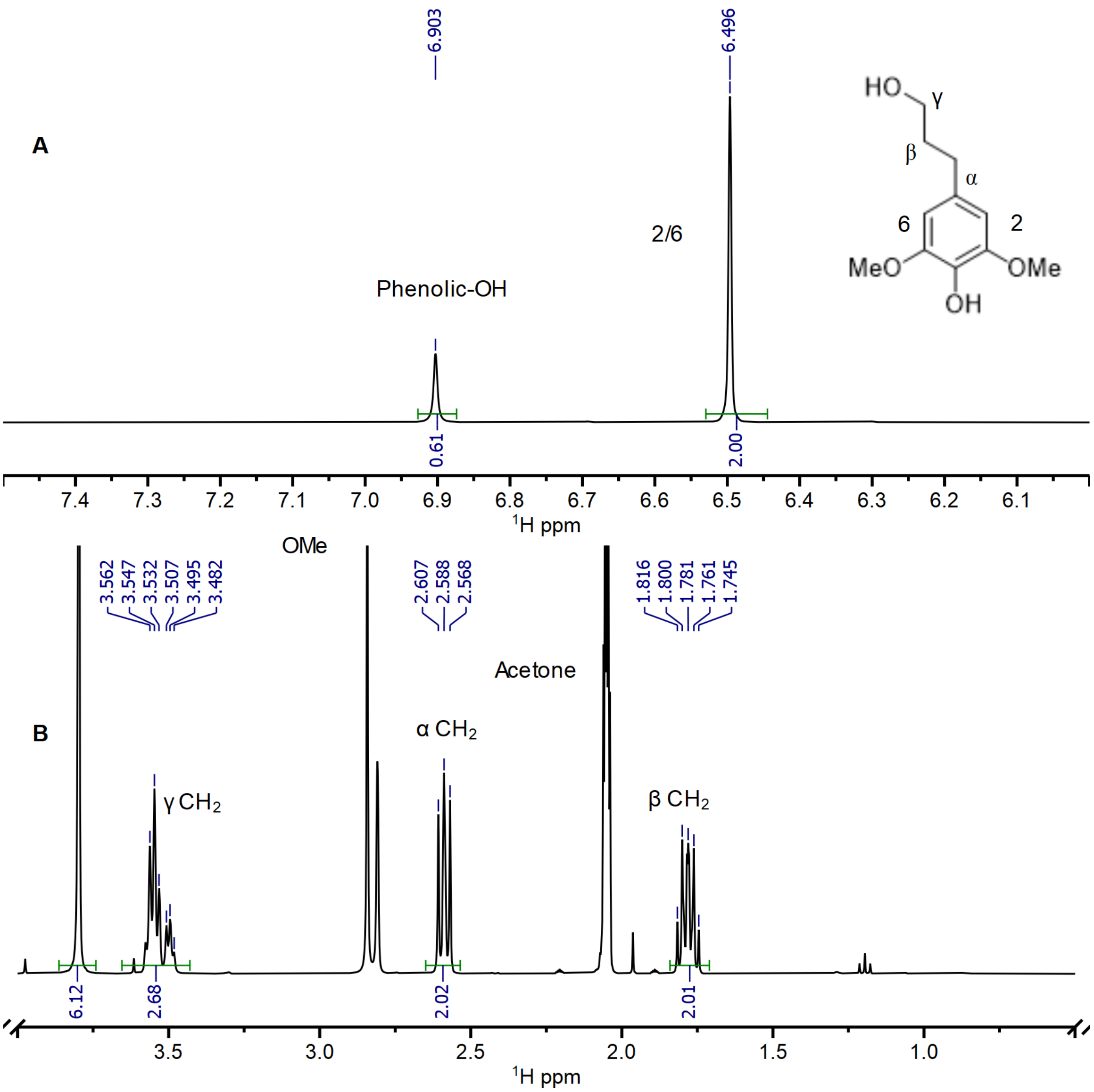

Figure S8. ¹H NMR spectrum of 4-(3-hydroxypropyl)-syringol (PSOH). (**A**) aromatic region. (**B**) Aliphatic region. ¹H NMR, acetone-d_6, 400 MHz.

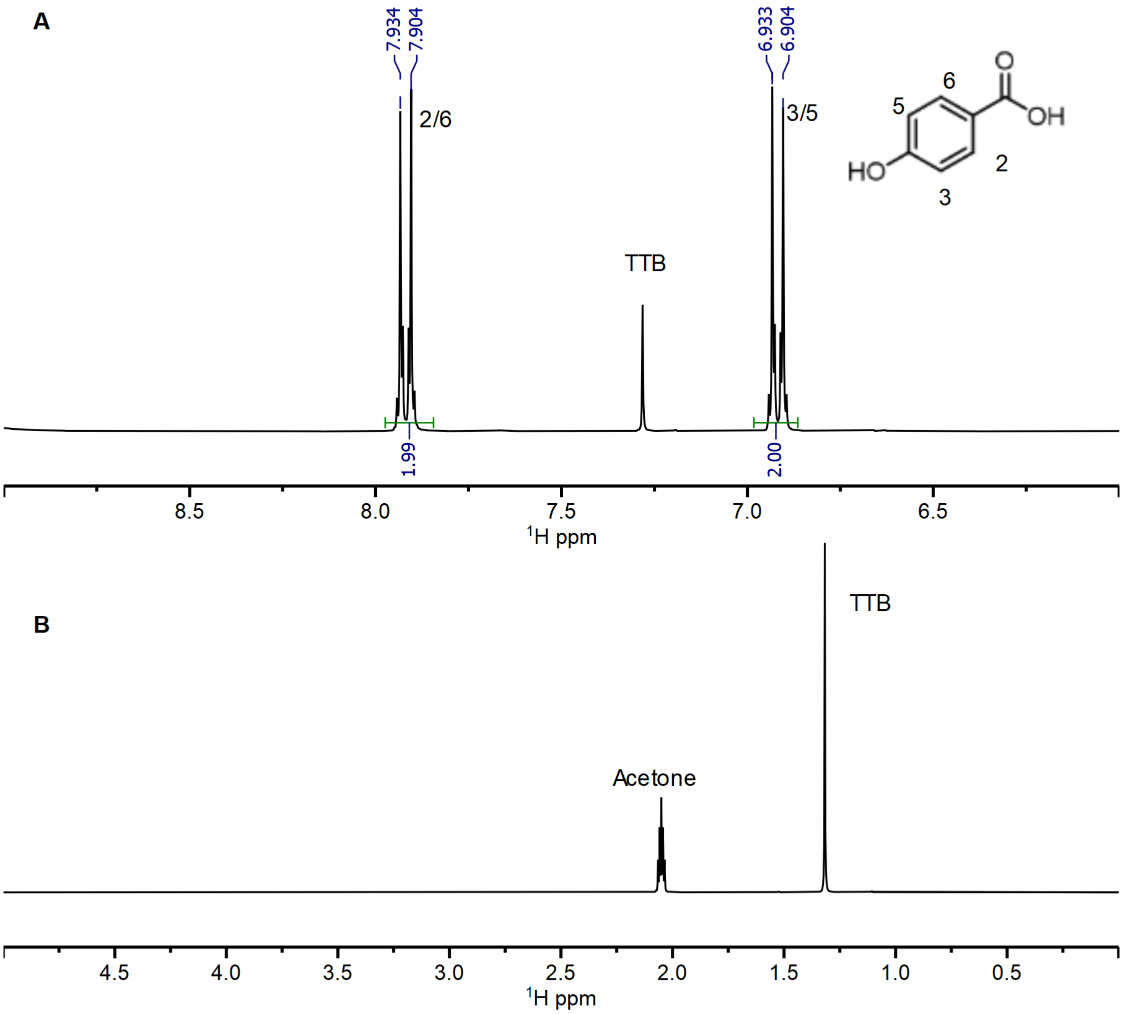

Figure S9. ^{1}H NMR spectrum of *para*-hydroxybenzoic acid (*p*-HBA). (**A**) aromatic region. (**B**) Aliphatic region. No upfield resonances were observed aside from acetone and TTB. ^{1}H NMR, acetone-d_6, 300 MHz.

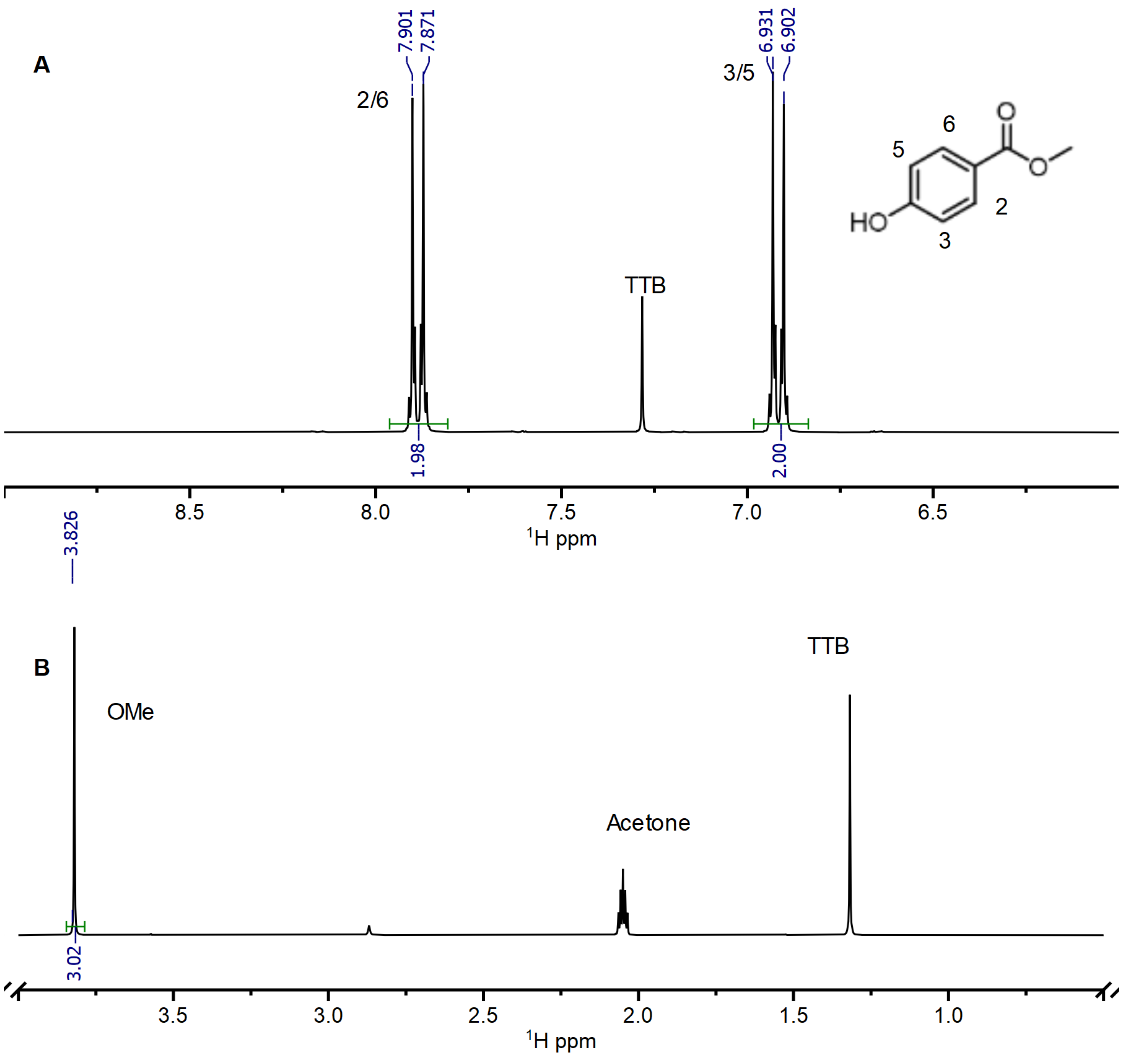

Figure S10. ^{1}H NMR spectrum of methyl paraben (MP). (**A**) aromatic region. (**B**) Aliphatic region. ^{1}H NMR, acetone-d_6, 300 MHz.

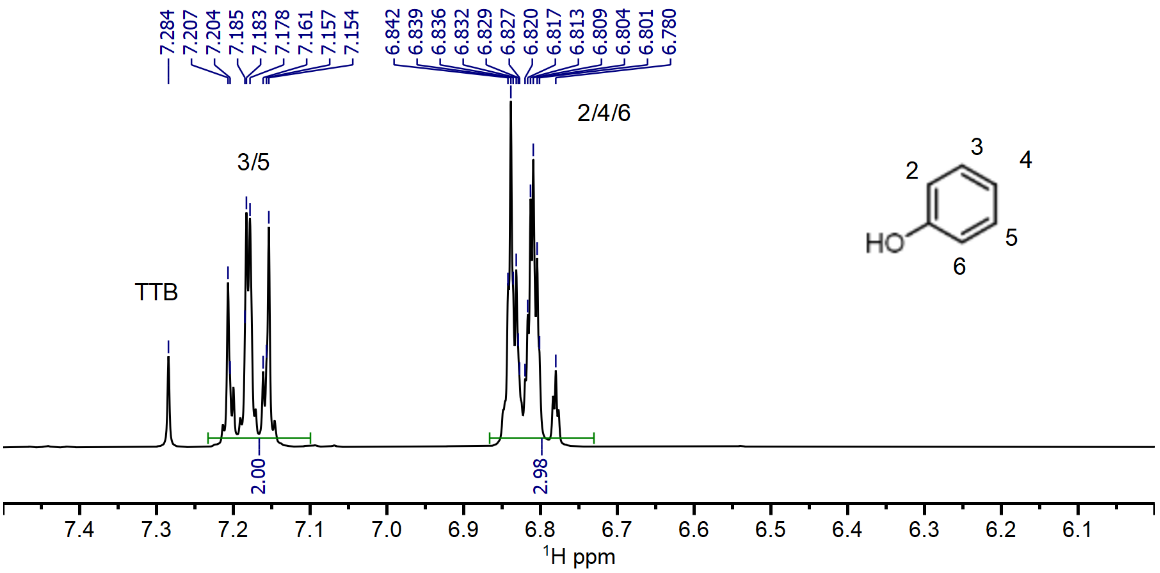

Figure S11. ¹H NMR spectrum of phenol. No upfield resonances were observed aside from acetone and TTB. ¹H NMR, acetone-d_6, 300 MHz.

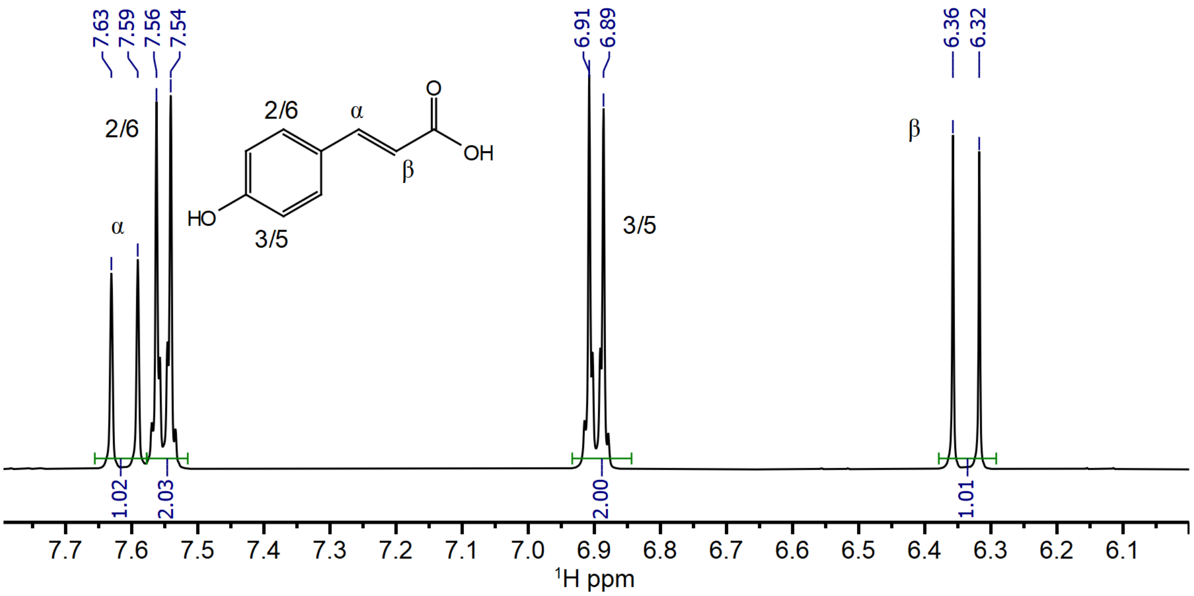

Figure S12. ^{1}H NMR spectrum of coumaric acid. No upfield resonances were observed aside from acetone and TTB. ^{1}H NMR, acetone-d_6, 400 MHz.

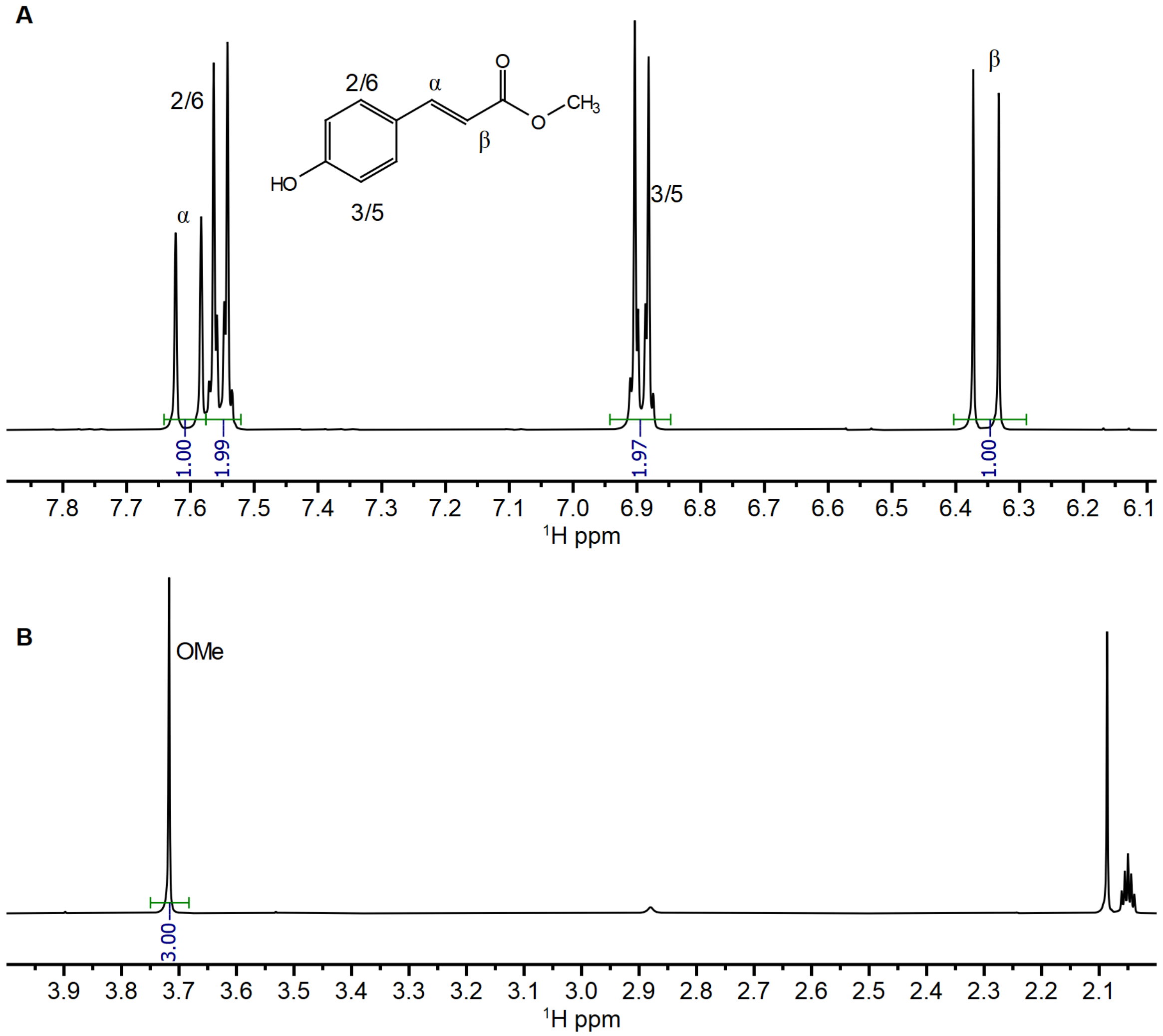

Figure S13. ^{1}H NMR spectrum of methyl coumarate. (**A**) aromatic region. (**B**) Aliphatic region. ^{1}H NMR, acetone-d_6, 400 MHz.

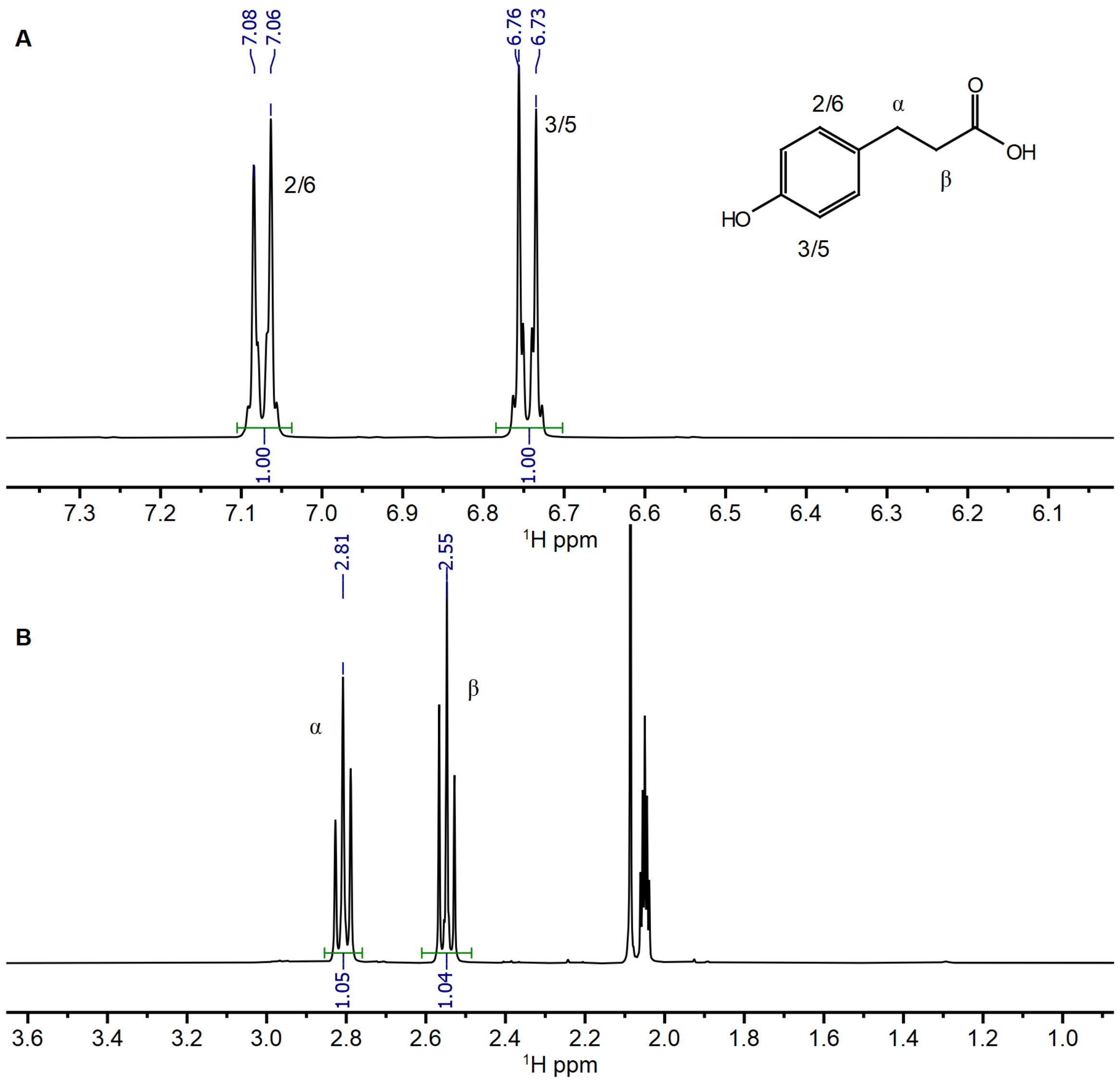

Figure S14. ^{1}H NMR spectrum of 3-(4-hydroxyphenyl)-propionic acid (**A**) aromatic region. (**B**) Aliphatic region. ^{1}H NMR, acetone-d_6, 400 MHz.

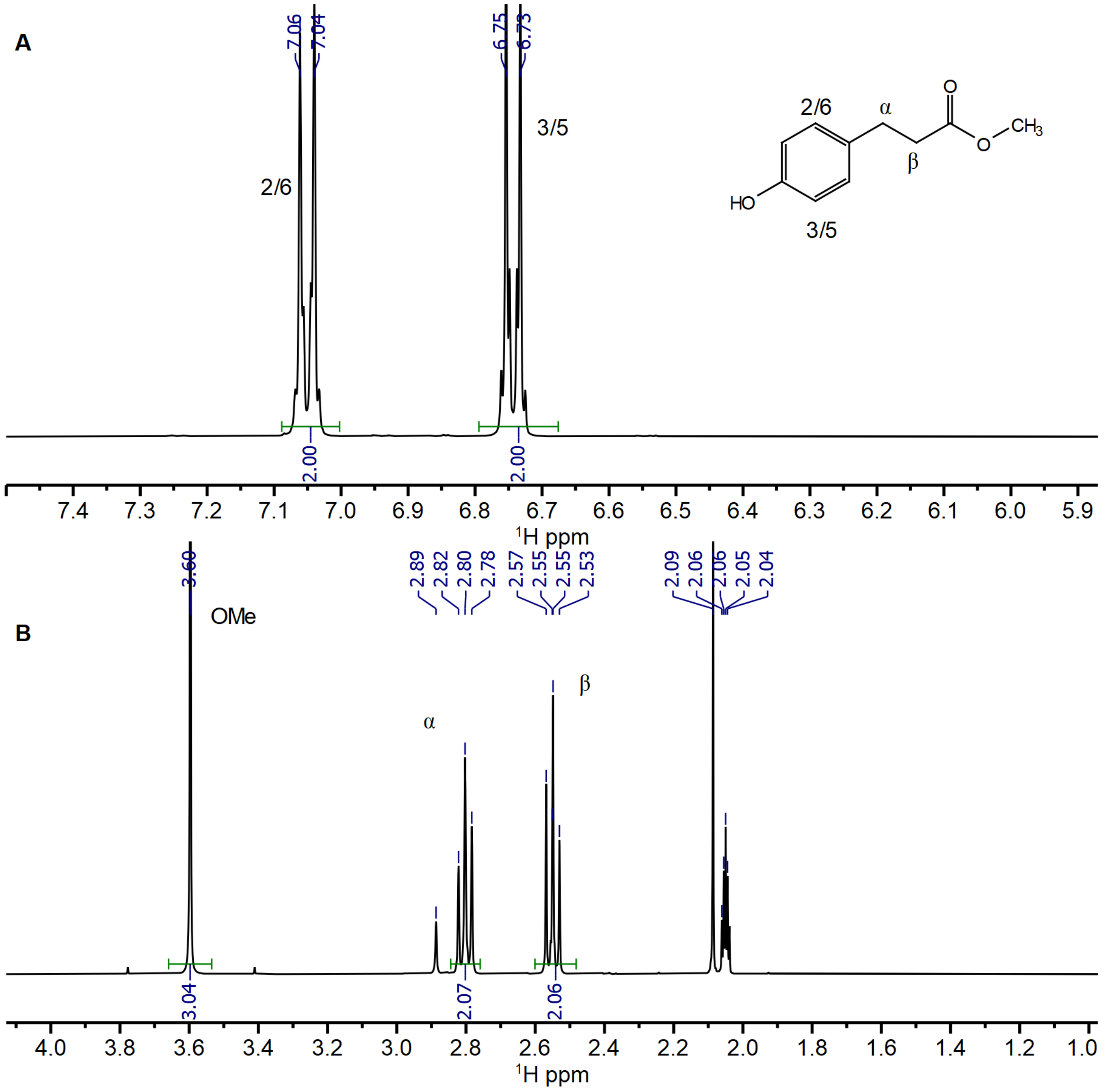

Figure S15. ^{1}H NMR spectrum of methyl-3-(4-hydroxyphenyl)-propionate (**A**) aromatic region. (**B**) Aliphatic region. ^{1}H NMR, acetone-d_6, 400 MHz.

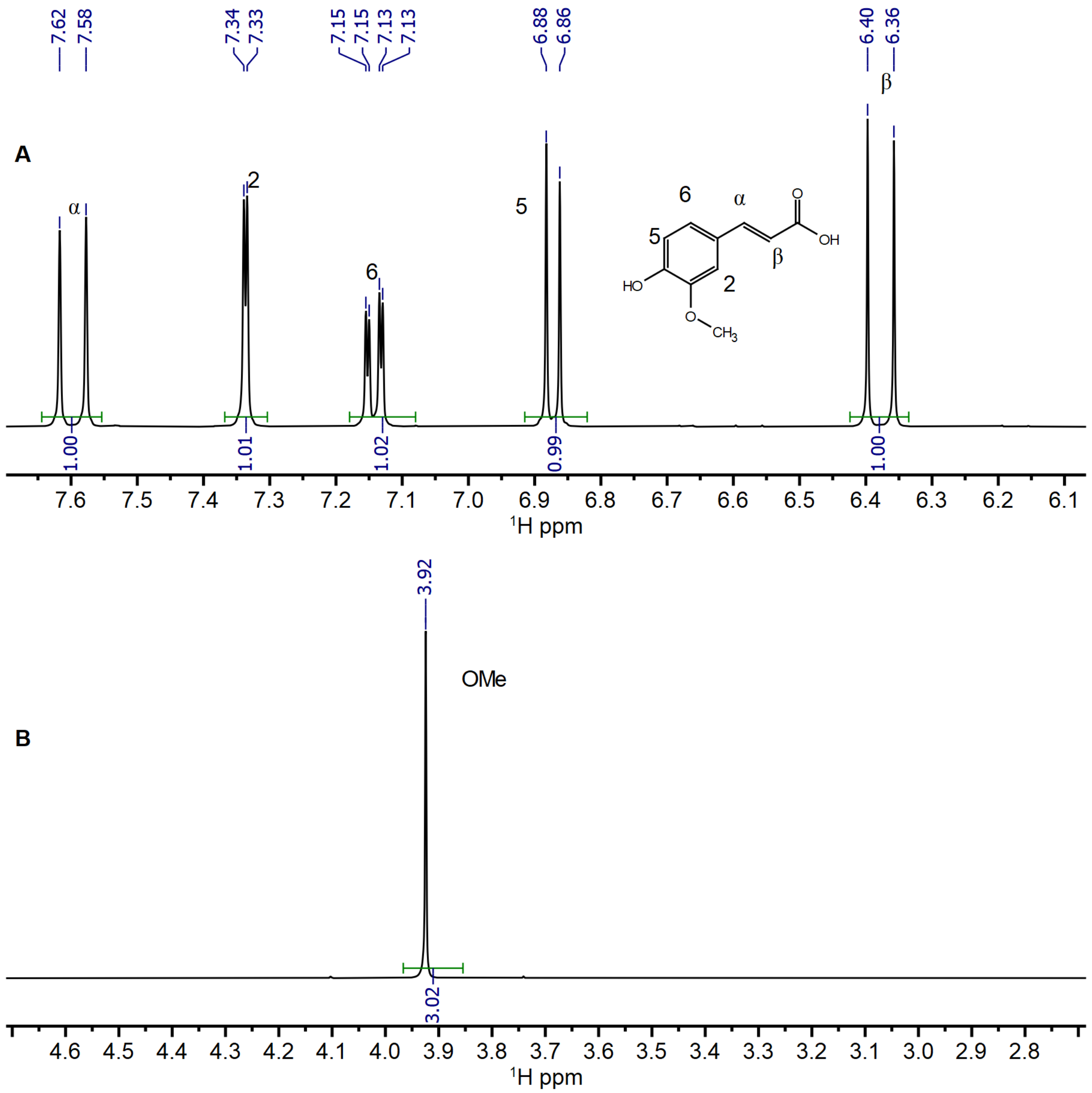

Figure S16. ^{1}H NMR spectrum of ferulic acid (**A**) aromatic region. (**B**) Aliphatic region. ^{1}H NMR, acetone-d_6, 400 MHz.

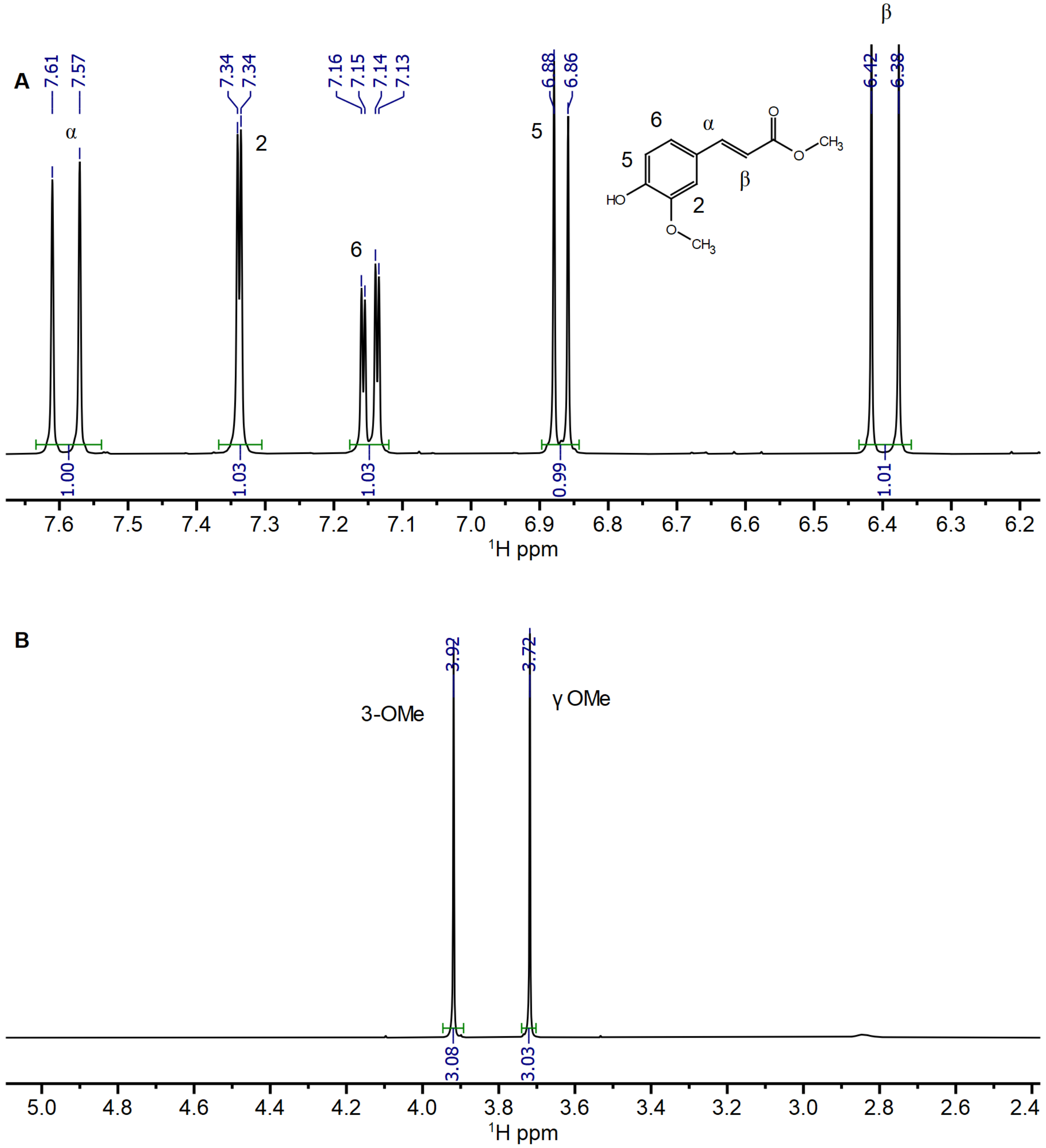

Figure S17. ¹H NMR spectrum of methyl ferulate (**A**) aromatic region. (**B**) Aliphatic region. ¹H NMR, acetone-*d*₆, 400 MHz.

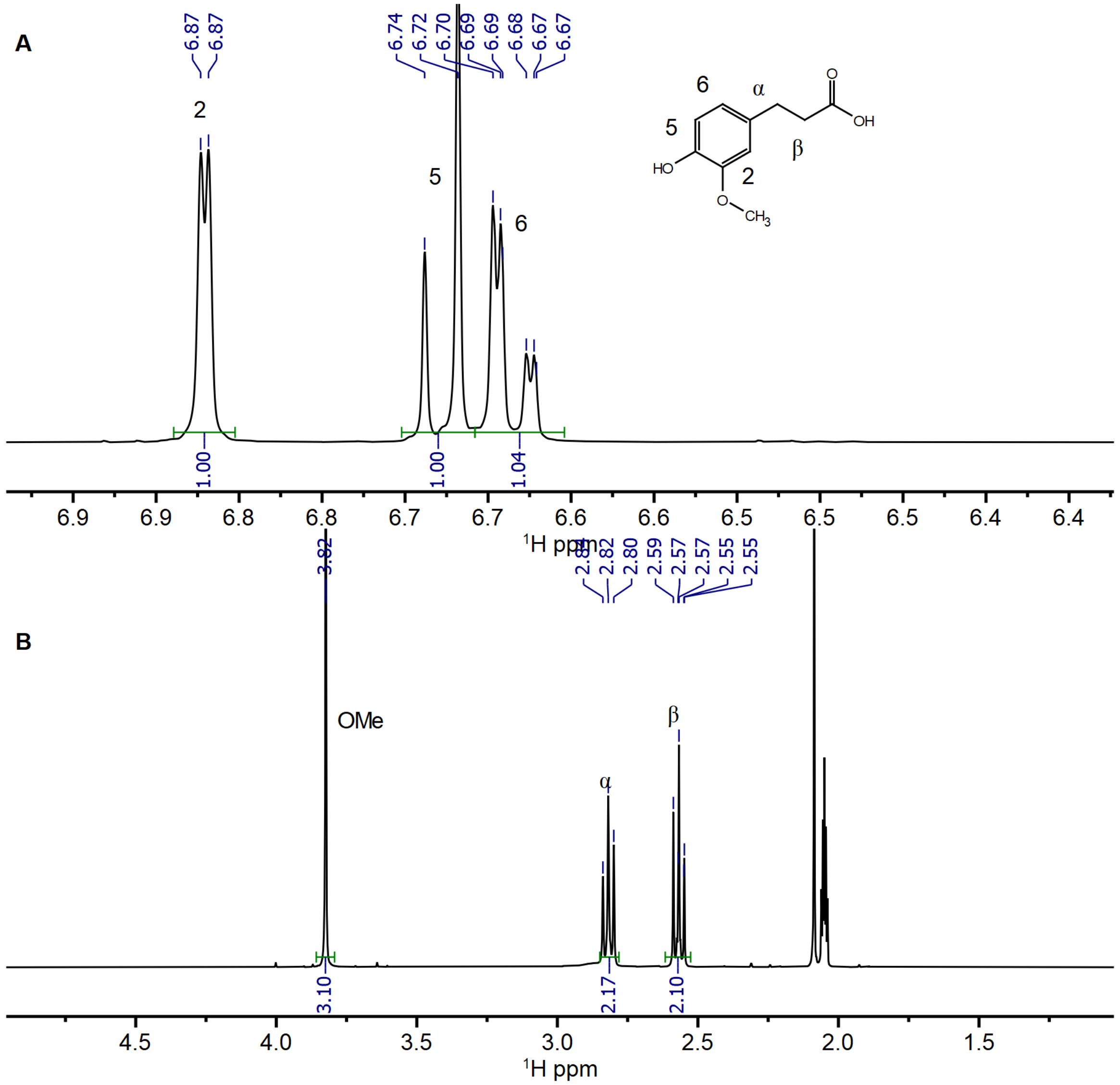

Figure 18. ^{1}H NMR spectrum of 3-(4-Hydroxy-3-methoxyphenyl)-propionic acid. (**A**) aromatic region. (**B**) Aliphatic region. acetone-d_6, ^{1}H NMR, acetone-d_6, 400 MHz.

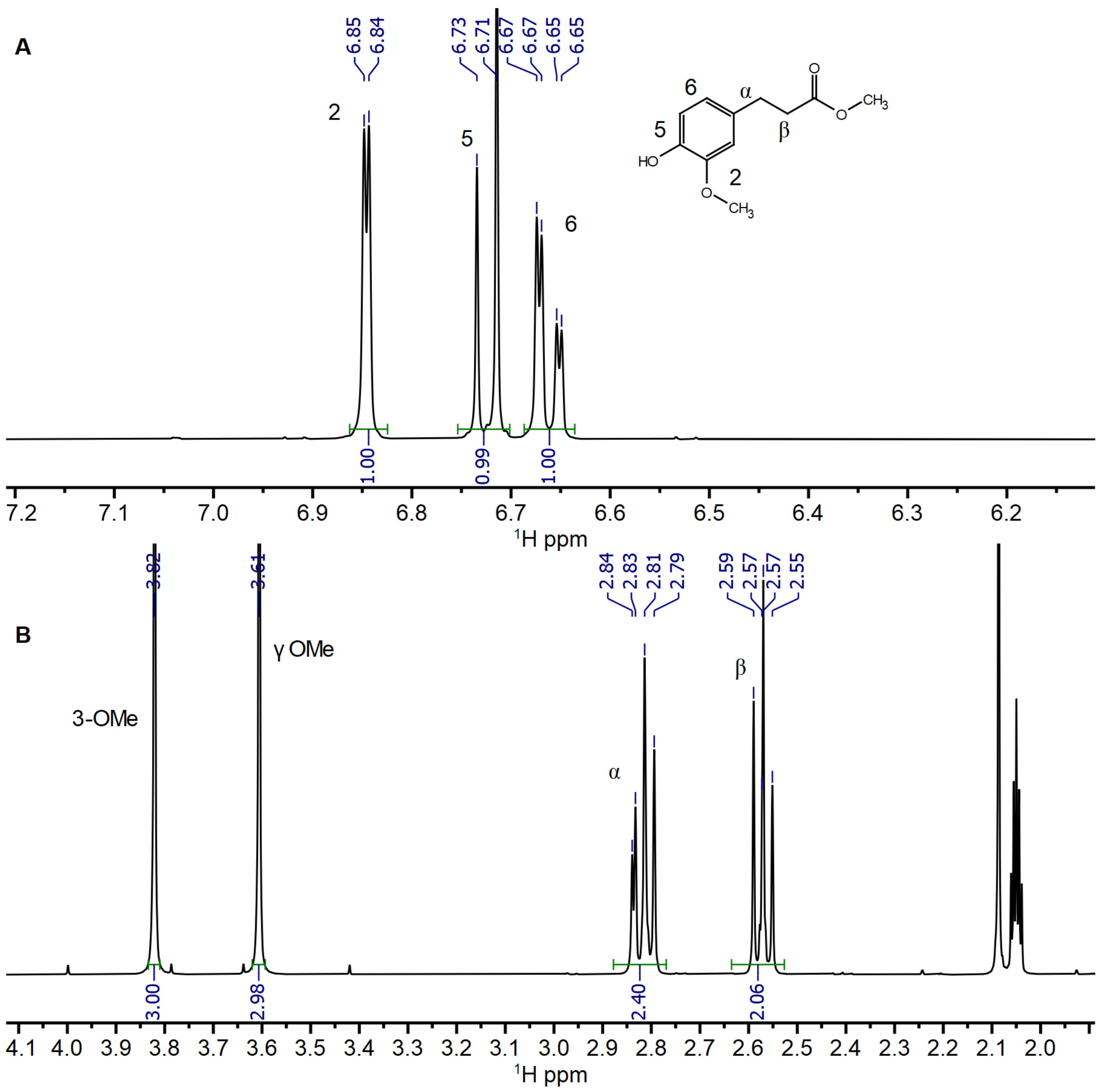

Figure S19. ^{1}H NMR spectrum of methyl 3-(4-hydroxy-3-methoxyphenyl)-propanoate. (**A**) aromatic region. (**B**) Aliphatic region. ^{1}H NMR, acetone-d_6, 400 MHz.

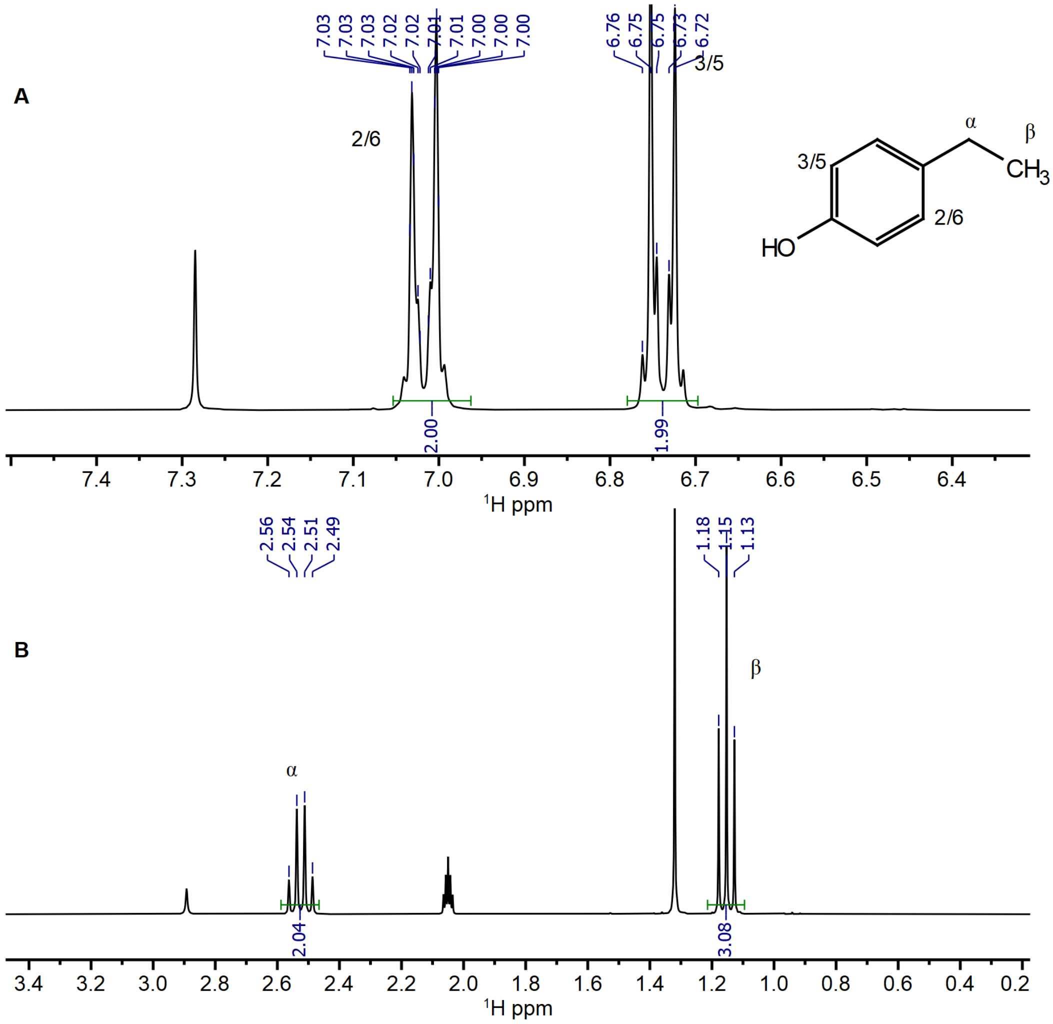

Figure S20. ¹H NMR spectrum of 4-ethylphenol (**A**) aromatic region. (**B**) Aliphatic region. ¹H NMR, acetone-d_6, 400 MHz.

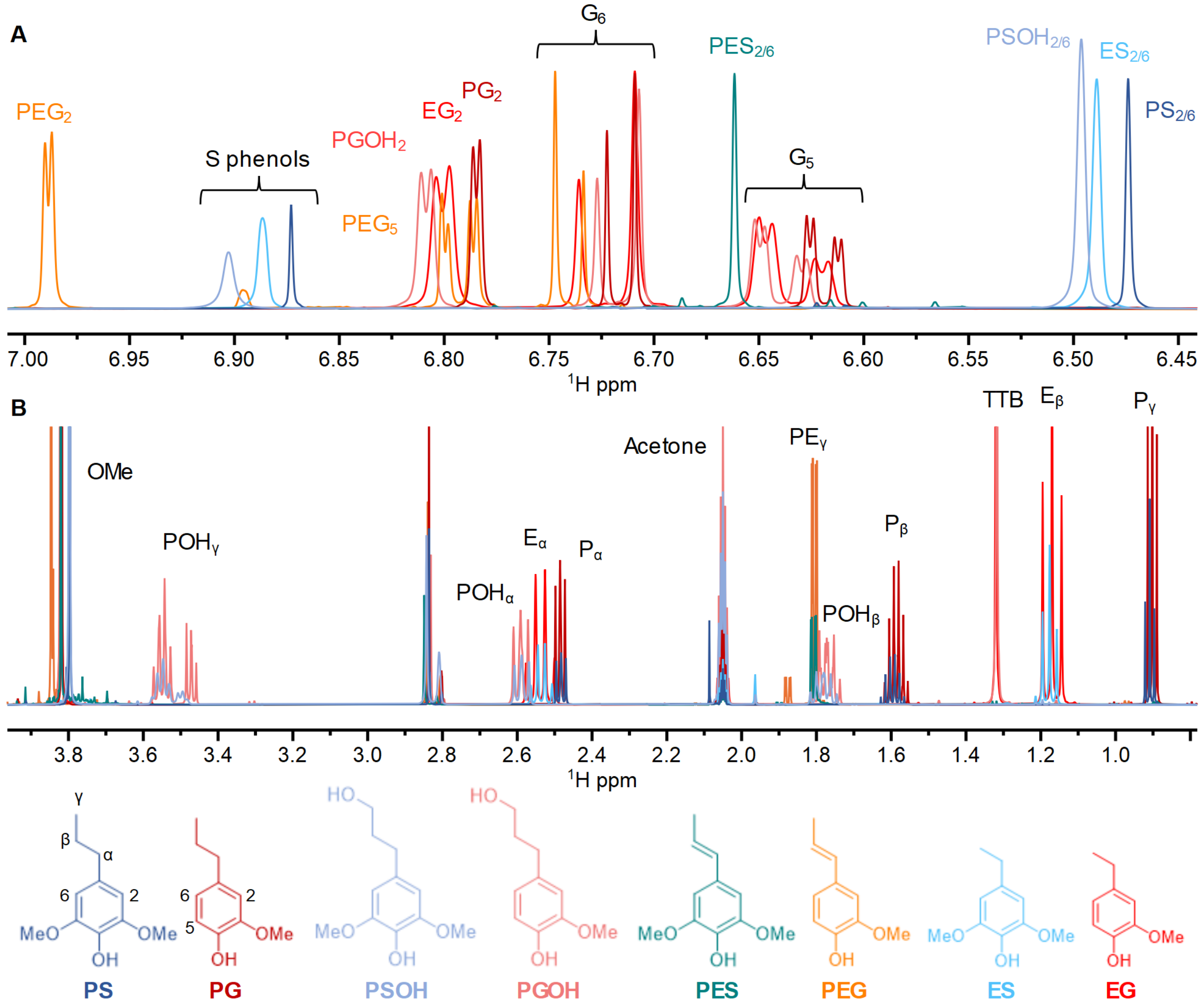

Figure S21. **^{1}H NMR spectra of RCF monomer model compounds. (A)** Aromatic region. G_5 refers to the G_5 proton of PG, PGOH, and EG. For clarity, G phenol resonances (7.18-7.27 ppm) and the α/β protons of PES and PEG (6.05-6.30 ppm) are not shown. **(B)** Aliphatic region. Peaks are labeled by their side chain selectivity: P, 4-propyl; POH, 4-(3-hydroxypropyl); E, 4-ethyl; PE, 4-propenyl. ^{1}H NMR, acetone-d_6, 400 MHz. This figure was created by overlaying individual spectra of model compounds.

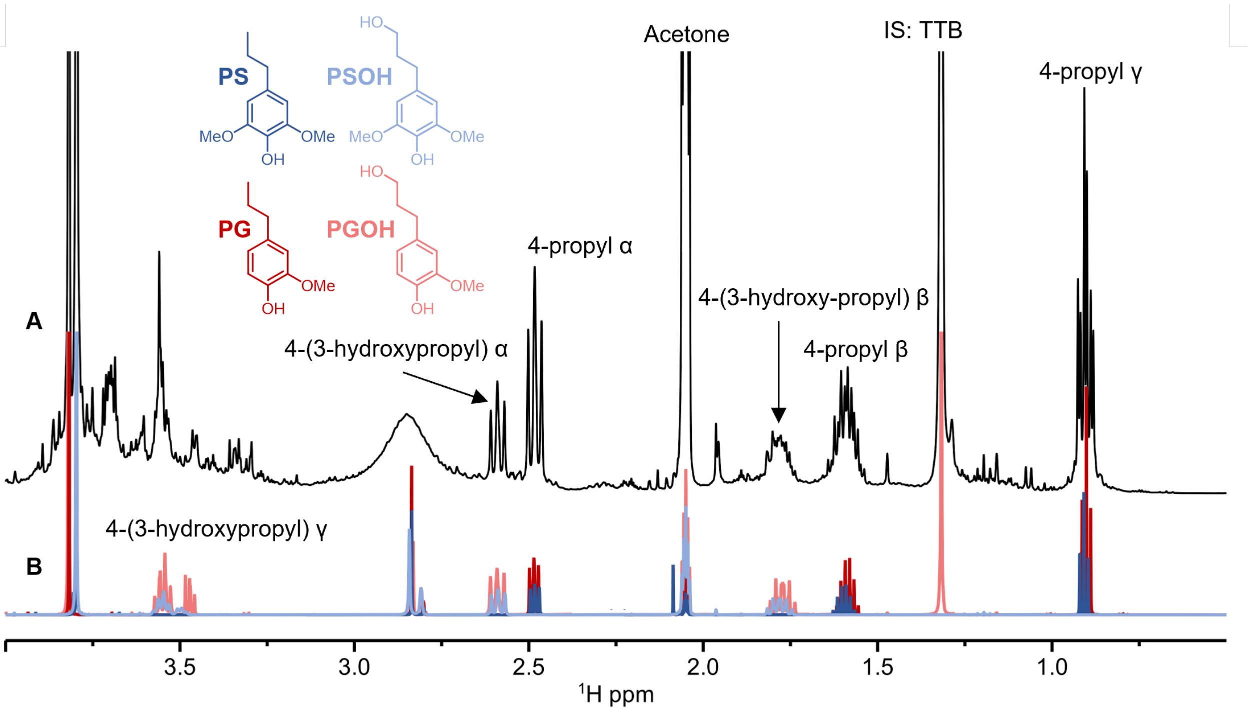

Figure S22. ¹H NMR spectrum, highlighting the aliphatic region of RCF oil produced using Ru/C leading to high selectivity in 4-propyl products with minor contributions from 4-(3-hydroxypropyl) products. (A) RCF oil and **(B)** 4-propyl and 4-(3-hydroxypropyl) model compound spectra. This figure is the aliphatic region of the same spectra shown in **Figure 1** in the main text. RCF conditions: 2 g poplar, 400 mg Ru/C (5 wt% Ru), 200 °C, 30 bar H_2, 3 h. The reaction product was filtered directly from the reactor and liquid-liquid extraction was not performed prior to NMR analysis. NMR specifications: 0.5 mL of reaction filtered liquor dried under flowing N_2 and dissolved in 1 mL 10:1 acetone-d_6/methanol-d_4 with 1 mg/mL 1,3,5-tri-*tert*-butylbenzene (TTB) as the internal standard. The methanol-d_4 was required to fully solubilize the unextracted sample. 32 scans, 3 s delay (d1), 0.3 Hz line broadening, and zero filling to 2x spectrum size. Monomers were identified by comparing spectra of authentic standards. ¹H NMR, acetone-d_6, 400 MHz.

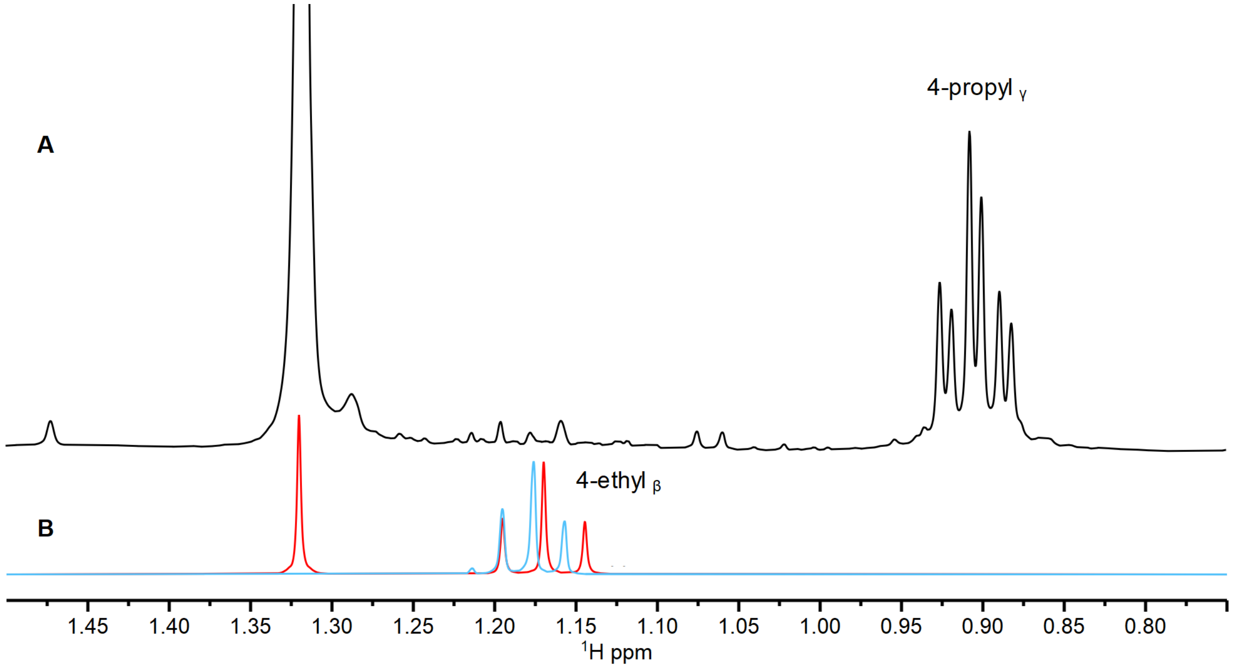

Figure S23. Aliphatic region showing low amounts of 4-ethylsyringol and 4-ethylguaiacol. (A) RCF oil spectrum. **(B)** 4-ethyl substituted monomers. This region can be used to rule out the substantial presence of 4-ethyl-substituted monomers. RCF

conditions: 2 g poplar, 400 mg Ru/C (5 wt% Ru), 3 h. 200 °C. The reaction product was filtered directly from the reactor and liquid-liquid extraction was not performed prior to NMR analysis. NMR specifications: 0.2 mL of reaction filtered liquor dried under flowing N_2 and dissolved in 1 mL 10:1 acetone-d_6/methanol-d_4 with ~ 1 mg/mL 1,3,5-tri-*tert*-butylbenzene (TTB) as the internal standard. The methanol-d_4 was required to fully solubilize the unextracted sample. 32 scans, 3 s delay (d1), 0.3 Hz line broadening, zero filling to 2x spectrum size. ^{1}H NMR, acetone-d_6 , 400 MHz.

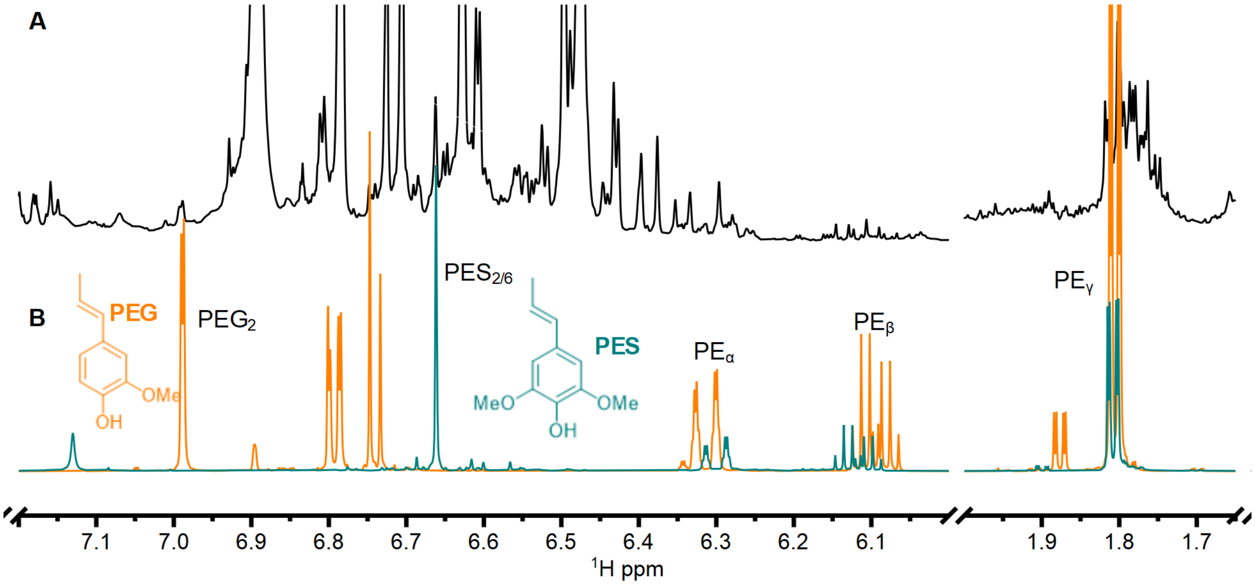

Figure S24. Example of low but quantifiable PES and PEG content in RCF oil. (**A**) RCF oil spectrum. (**B**) NMR spectra of isoeugenol and 4-propenylsyringol. RCF conditions: 2 g poplar, 400 mg Ru/C, 200 °C, 30 mL methanol, 3 h, 30 bar H_2 at room temperature. Liquid-liquid extraction performed with ethyl acetate. NMR specifications: 0.2 mL of extracted RCF oil solution in methanol (27 mg/mL) dried under flowing N_2 and dissolved in 1 mL acetone-d_6 with ~ 1 mg/mL TTB as the internal standard. 32 scans, 3 s delay (d1), 0.3 Hz line broadening, zero filling to 2x spectrum size. ^{1}H NMR, acetone-d_6 , 400 MHz.

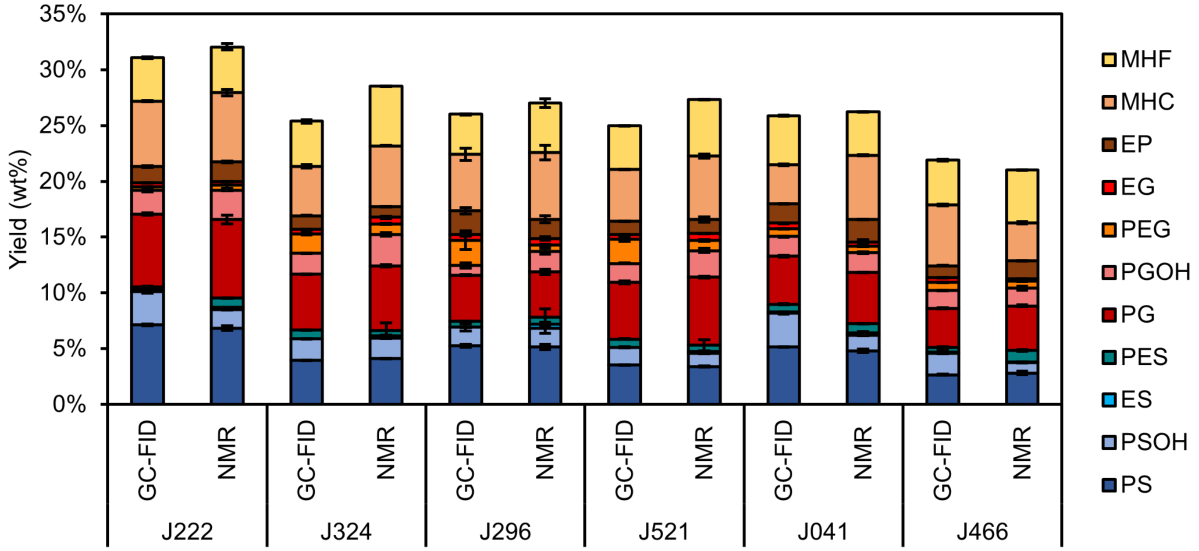

Figure S25. Comparison of quantifications of switchgrass samples between the NMR method and GC-FID. The GC-FID monomer quantifications and oil yield quantifications from NMR were presented in our previous work.[1] Error bars indicate the range of duplicate RCF experiments. RCF conditions: 2 g biomass, 400 mg Ru/C, 225 °C, 30 bar H_2, 3 h. NMR specifications: 0.5 mL of extracted oil dried under flowing N_2 and dissolved in 1 mL 10 acetone-d_6 with ~ 1 mg/mL TTB as the internal standard. 32 scans, 3 s delay (d1), 0.3 Hz line broadening, zero filling to 2x spectrum size.

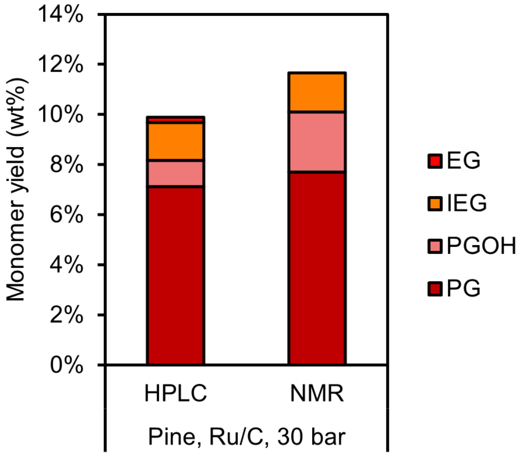

Figure S26. Comparison of quantifications of a pine sample between the NMR method and GC-FID. RCF conditions: 2 g biomass, 400 mg Ru/C, 225 °C, 30 bar H_2, 3 h, LLE with ethyl acetate/water. NMR specifications: 0.2 mL of extracted oil dried under flowing N_2 and dissolved in 1 mL 10 acetone-d_6 with ~ 1 mg/mL TPA as the internal standard. 32 scans, 30 s delay (d1), 0.3 Hz line broadening, one level of zero filling.

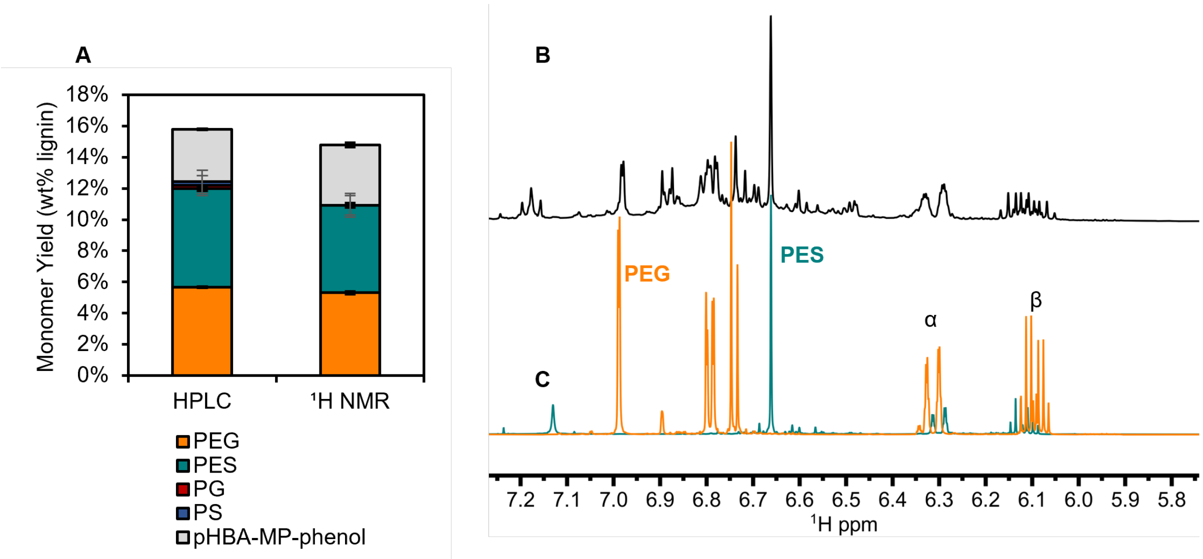

Figure S27. RCF reactions under H_2-free conditions using Ru/C leading to high selectivity in PEG and PES. (A) Comparison of quantifications obtained with UHPLC versus the ^{1}H NMR method. **(B)**NMR spectrum of the RCF oil. **(C)** NMR spectra of model compounds isoeugenol (PEG) and 4-proepnylsyringol (PES), illustrating large peaks attributed to the PEG and PES in the RCF oil. ^{1}H NMR, acetone-d_6, 400 MHz. Other lignin monomer products were not quantified in ^{1}H NMR, however small (<0.5 wt%) of 4-propylsyringol and 4-propylguaiacol were measured via UHPLC. Error bars represent the standard deviation of three replicate measurements (replicate dilutions for UHPLC and replicate NMR samples for NMR) of a single sample. RCF conditions: six identical reactions were performed with the following conditions and combined in the workup to produce a single RCF oil sample which was measured three times for HPLC and NMR: 2 g poplar, 100 mg Ru/C (5 wt% Ru), 225 °C, 0 bar H_2, 3 h. Liquid-liquid extraction was performed with ethyl acetate and water to isolate the RCF oil for gravimetric measurements. NMR specifications: 0.2 mL of extracted oil dried under flowing N_2 and dissolved in 1 mL 10 acetone-d_6 with ~ 1 mg/mL TPA as the internal standard. 32 scans, 30 s delay (d1), 0.3 Hz line broadening, zero filling to 2x spectrum size.

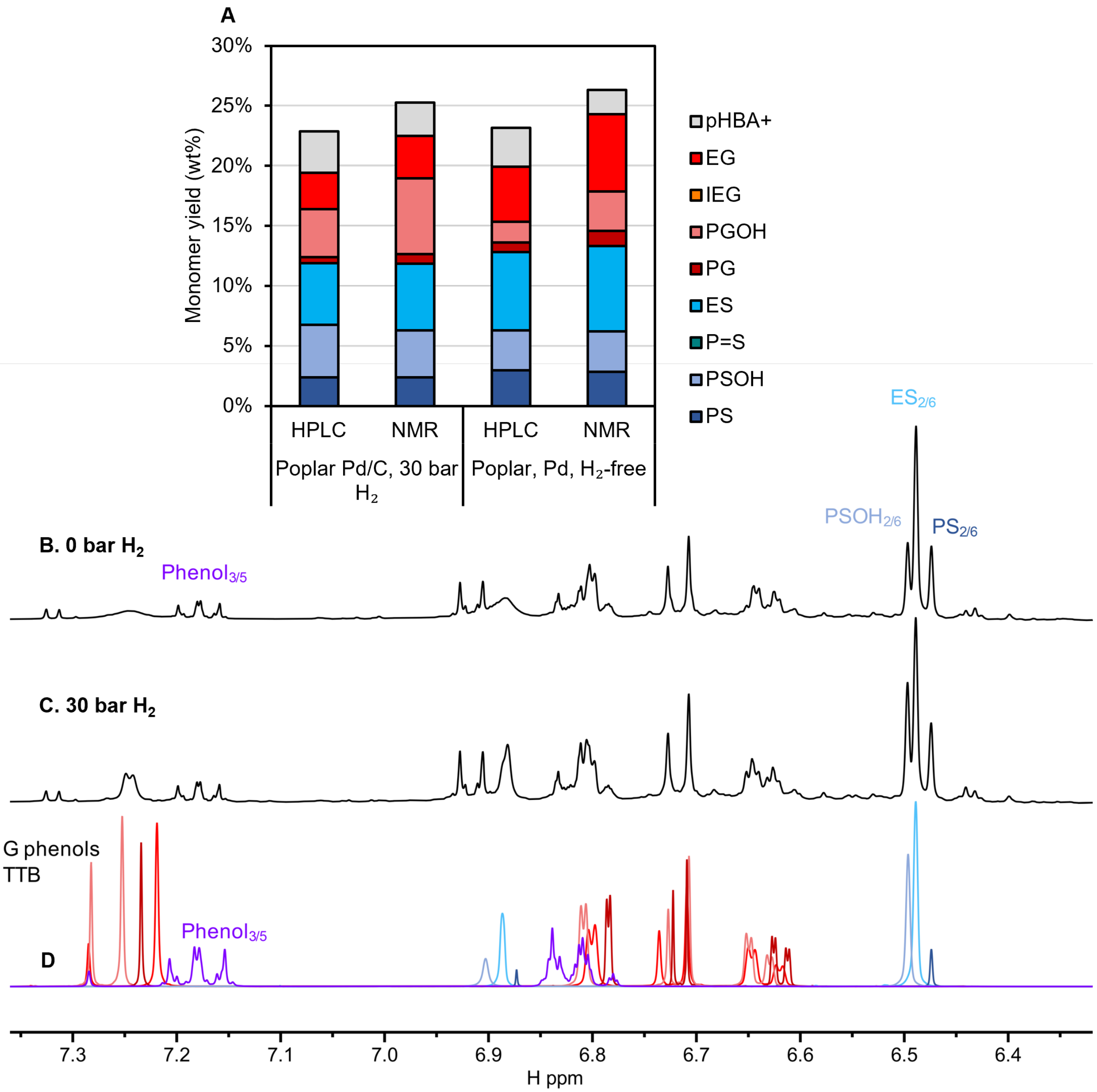

Figure S28. **Quantifications of RCF reactions using Pd/C with and without H_2. (A)** Quantification comparison of ^{1}H NMR and UHPLC. G monomers are overquantified due to the high degree of overlap with PGOH and EG, as well as the presence of phenol. 4-(3-hydroxylpropyl)-guaiacol was quantified from its β resonance by subtracting out the contribution from 4-(3-hydroxylpropyl)-syringol. 4-ethylguaiacol and 4-propylguaiacol were quantified via peak deconvolution of their β and γ proton resonances respectively. **(B)** RCF oil spectrum without added H_2. **(C)** RCF oil spectrum with 30 bar added H_2. **(D)** Model compound spectra. RCF conditions: 2 g biomass, 200 mg Ru/C, 225 °C, 30 or bar H_2, 3 h, LLE with ethyl acetate/water. NMR specifications: 0.2 mL of extracted oil dried under flowing N_2 and dissolved in 1 mL 10 acetone-d_6 with ~ 1 mg/mL TPA as the internal standard. 32 scans, 3 s delay (d1), 0.3 Hz line broadening, zero filling to 2x spectrum size.

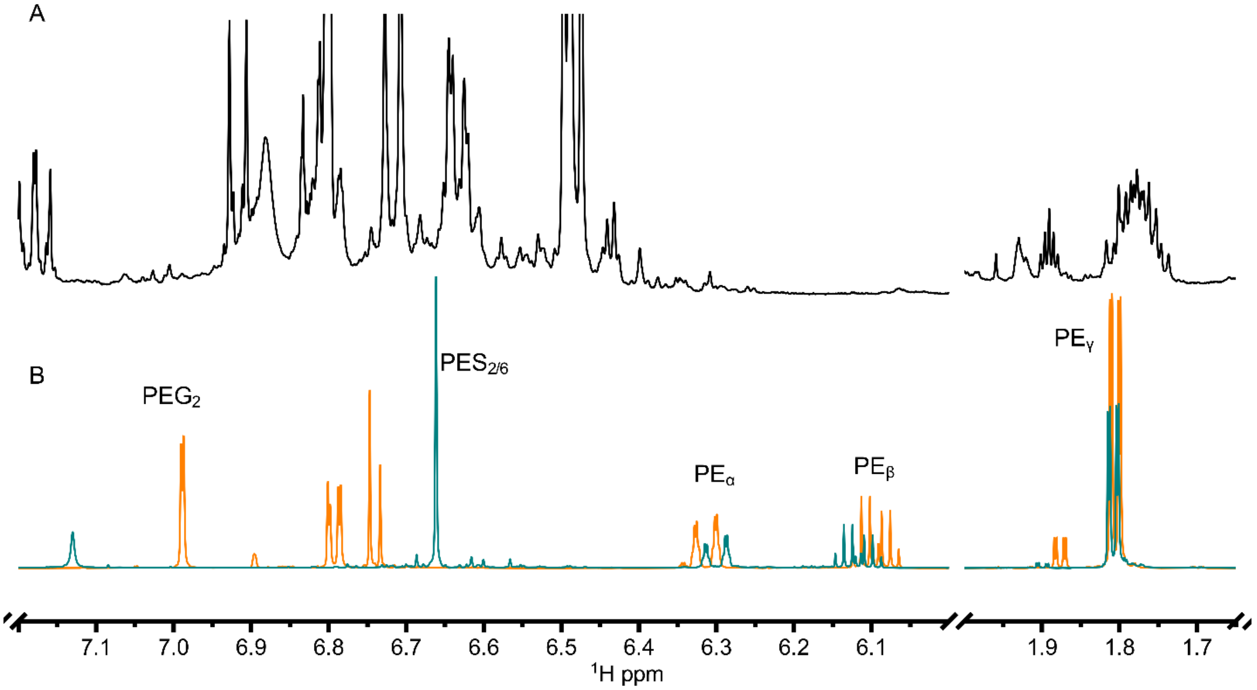

Figure S29. Comparison of ^{1}H NMR spectra of RCF oil demonstrating the lack of PES and PEG. A) RCF oil generated using Pd/C under H_2-free conditions, leading to intermediate selectivity in 4-propyl, 4-ethyl,and 4-(3-hydroxypropyl) products. RCF conditions: 2 g poplar, 200 mg Pd/C, 225 °C, 30 mL methanol, 3 h, 1 bar He at room temperature. Liquid-liquid extraction performed with ethyl acetate. B) Reference spectra of PEG and PES. NMR specifications: 0.2 mL of RCF oil solution (34 mg extracted oil/mL) liquor dried under flowing N_2 and dissolved in 1 mL 1 acetone-d_6 with ~ 1 mg/mL terephthalaldehyde (TPA) as the internal standard. The 32 scans, 30 s delay (d1), 0.3 Hz line broadening, zero filling to 2x spectrum size.

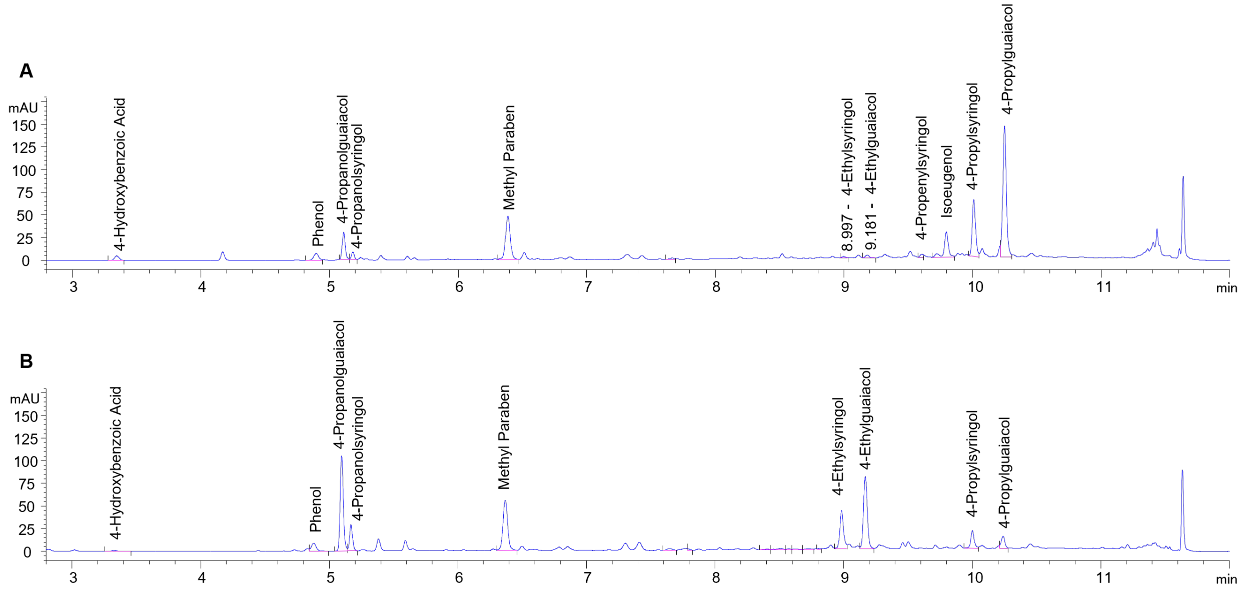

Figure S30. Example chromatograms from the UHPLC method. **(A)**Ru/C with 30 bar H_2. **(B)** Pd/C with 30 bar H_2.

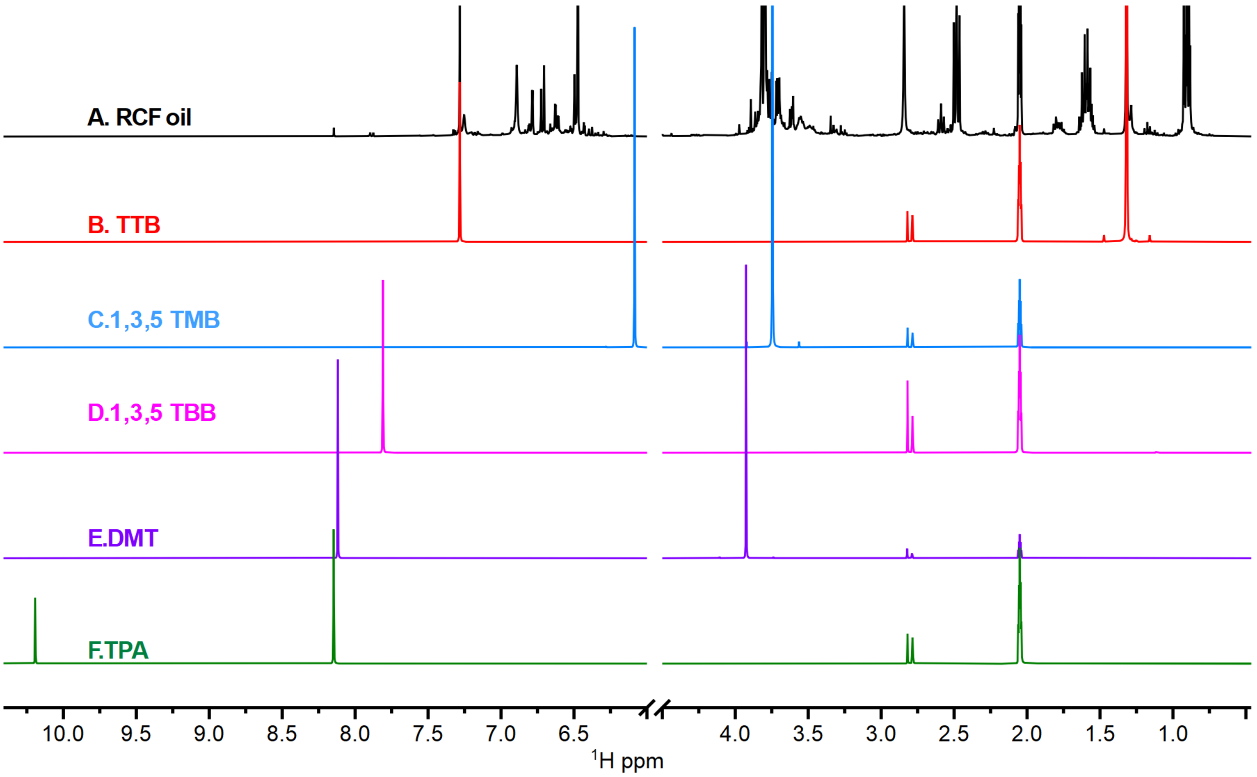

Figure S31. Comparison of ^{1}H NMR spectra of RCF oil with various choices of internal standards. (A) RCF oil. RCF conditions: 2 g biomass, 400 mg Ru/C, 200 °C, 30 bar H_2, 3 h, LLE with ethyl acetate/water. The RCF oil spectrum has TTB added as an internal standard. **(B)** 1,3,5-tri-*tert*-butylbenzene (TTB) **(C)** 1,3,5-trimethoxybenzene (TMB) **(D)** 1,3,5-tribromobenzene (TBB) **(E)** Dimethyl terephthalate (DMT) **(F)** Terephthalaldehyde (TPA).

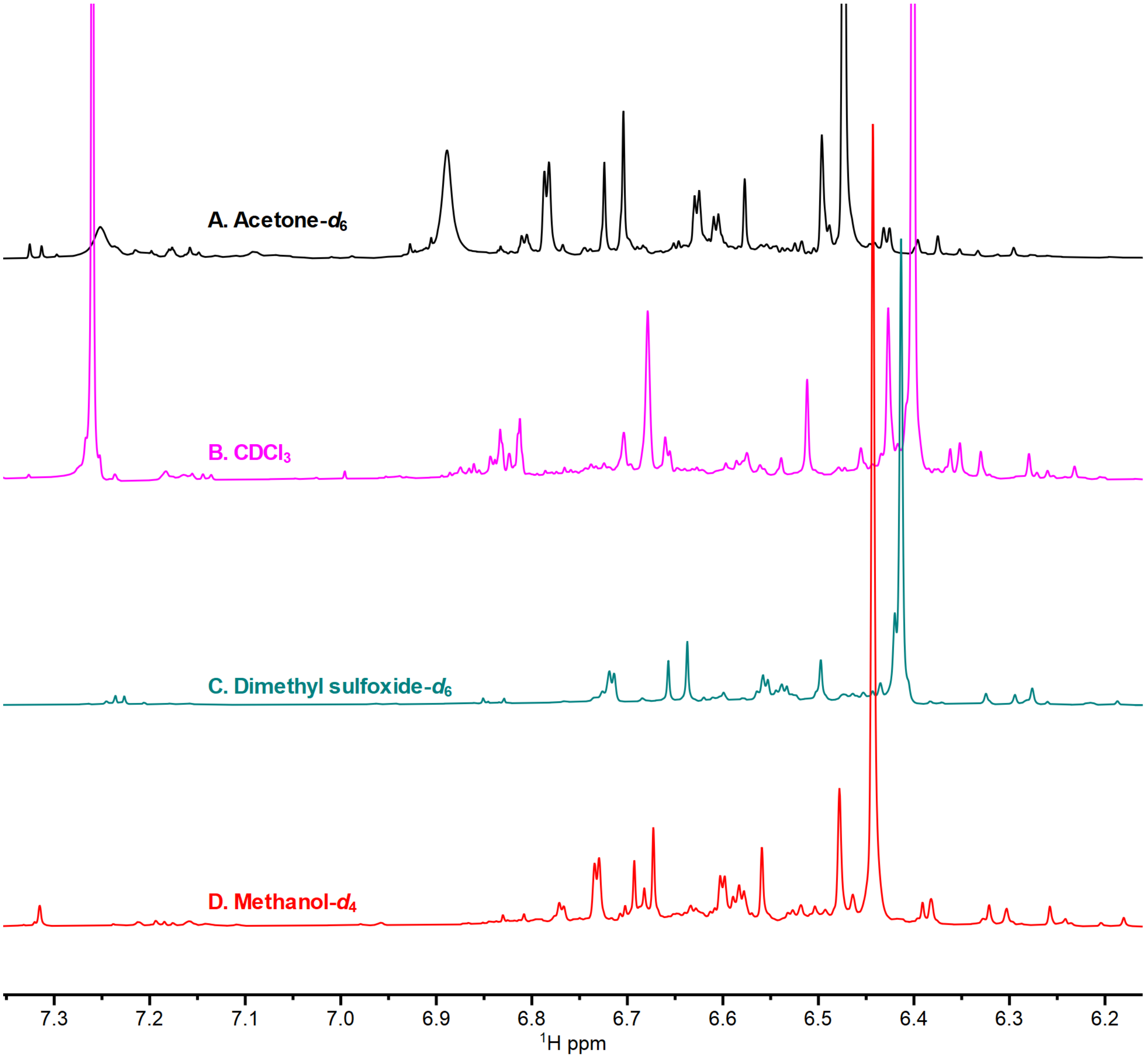

Figure S32. Comparison of RCF oil ^{1}H NMR spectra in different NMR solvents. **(A)** acetone-d_6 **(B)** $CDCl_3$ **(C)** Dimethyl sulfoxide-d_6 **(D)** Methanol-d_4. RCF conditions: 2 g biomass, 400 mg Ru/C, 200 °C, 30 bar H_2, 3 h, LLE with ethyl acetate/water. NMR specifications: 0.2 mL of extracted oil dried under flowing N_2 and dissolved in 1 mL 10 acetone-d_6 with ~ 1 mg/mL TTB as the internal standard. 32 scans, 3 s delay (d1), 0.3 Hz line broadening, zero filling to 2x spectrum size.

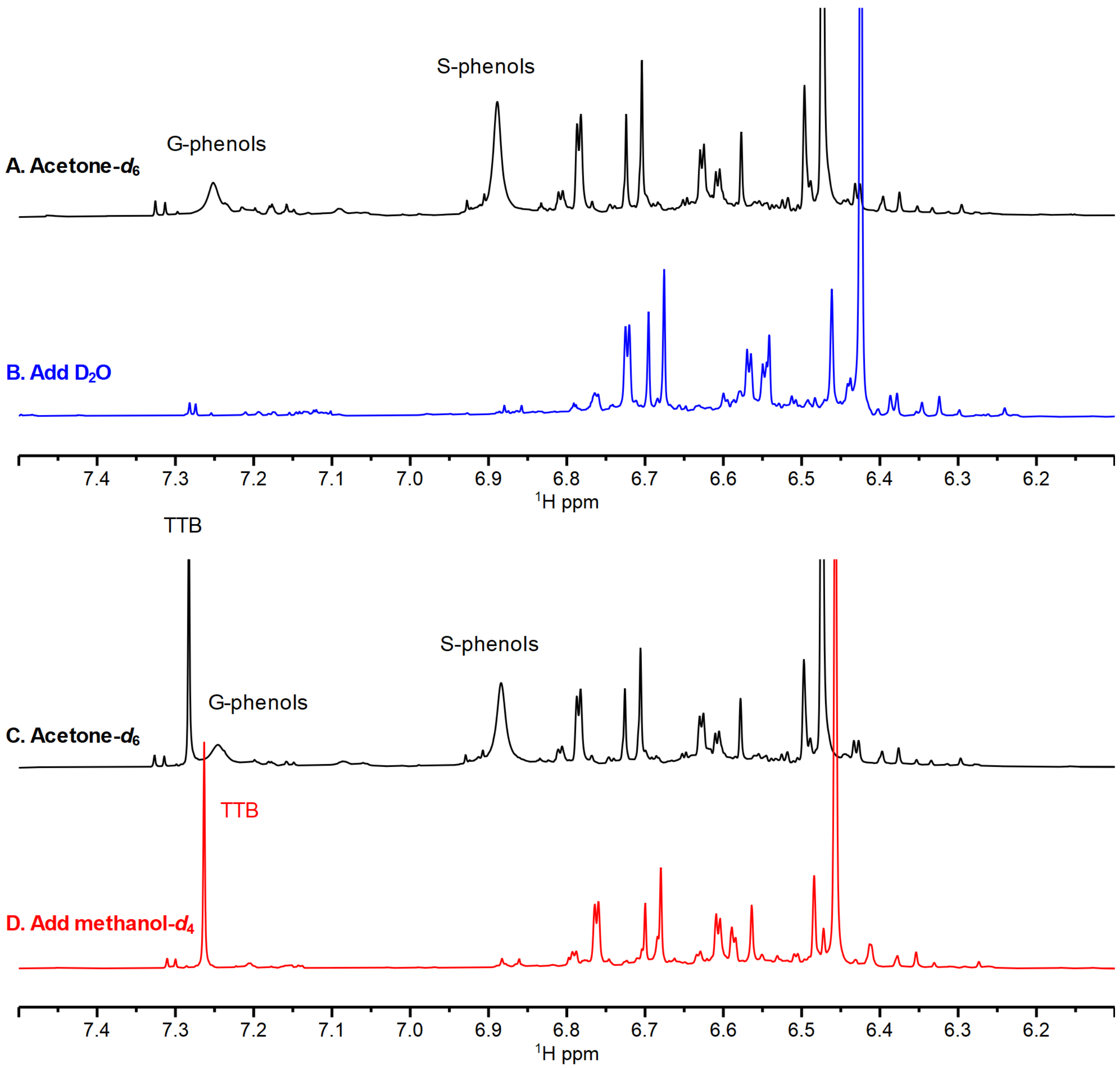

Figure S33. Identification of phenolic hydroxyl peaks by addition of D_2O or methanol-*d*$_4$. (**A**) RCF oil in acetone-*d*$_6$. (**B**) The RCF oil with 0.1 mL D_2O added. (**C**) RCF oil in acetone with TTB internal standard dissolved. (**D**) RCF oil with methanol-*d*$_4$ added. RCF conditions: 2 g biomass, 400 mg Ru/C, 200 °C, 30 bar H_2, 3 h, 30 mL MeOH, LLE with ethyl acetate/water. NMR specifications: 0.2 mL of extracted oil dried under flowing N_2 and dissolved in 1 mL 10 acetone-d_6 with ~ 1 mg/mL TTB as the internal standard (in the case of C, D; no TTB in A, B). 0.1 mL of the additional solvent was added to the NMR tube after the initial experiment. 32 scans, 3 s delay (d1), 0.3 Hz line broadening, zero filling to 2x spectrum size.

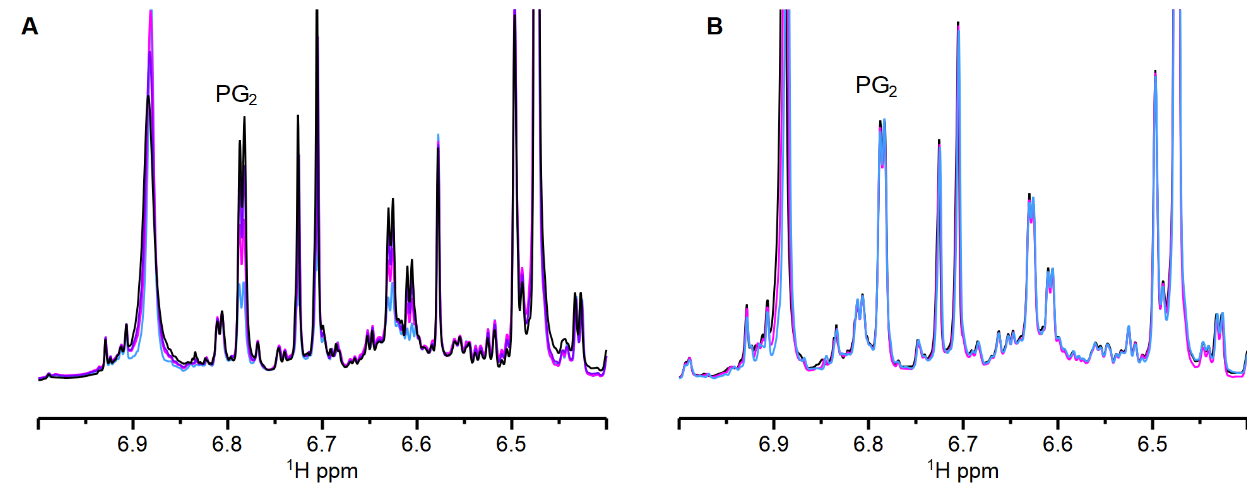

Figure S34. Example of over-drying resulting in the loss of 4-propylguaiacol. (A) Samples prepared by added varying amounts (0.1, 0.2, 0.3, 0.5 mL) of extracted RCF oil to the 4 mL vials for drying and drying under N_2 for 15 minutes. **(B)** Samples prepared by adding varying amounts of a different RCF oil (0.1, 0.25, 0.5 mL) to the 4 mL vial for drying and drying until the oil looked dry and it visually appeared that no solvent was in the vial. RCF conditions: 2 g biomass, 400 mg Ru/C, 225 °C, 30 bar H_2, 3 h, 30 mL MeOH, LLE with ethyl acetate/water. NMR specifications: 0.1-0.5 mL of extracted oil dried under flowing N_2 and dissolved in 1 mL 10 acetone-d_6 with ~ 1 mg/mL TTB as the internal standard. 32 scans, 3 s delay (d1), 0.3 Hz line broadening, zero filling to 2x spectrum size.

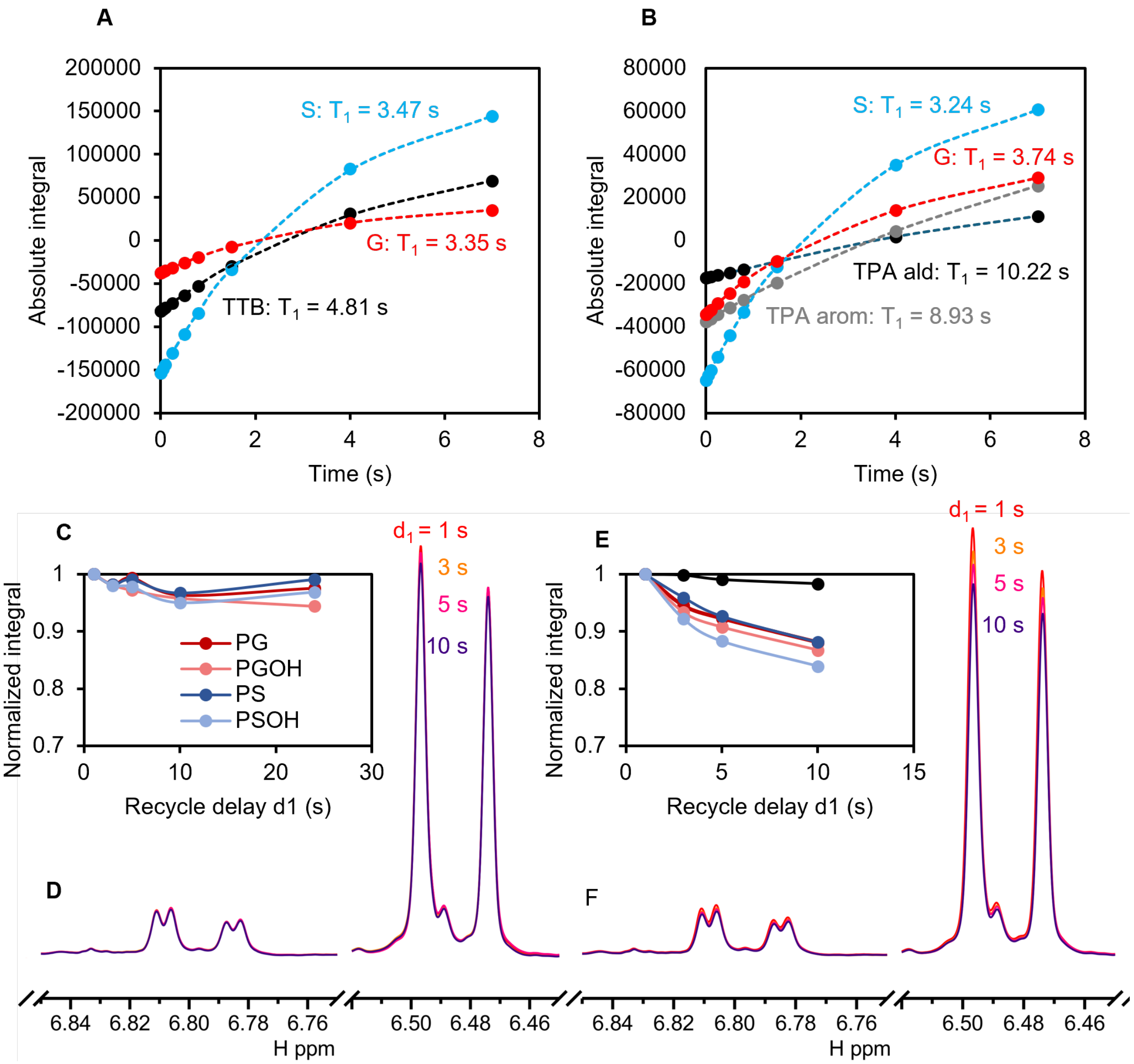

Figure S35. Measurement of relaxation characteristics of RCF oil and internal standards. (A) Inversion recovery experiment for RCF oil with 1,3,5-tri-*tert*-butylbenzene (TTB) as the internal standard. **(B)** Inversion recovery experiment for RCF oil with terephthalaldehyde (TPA) as the internal standard. **(C)** Normalized integrals for zg30 experiments run with different d1 parameters for TTB. **(D)** Normalized integrals for zg30 experiments run with different d1 parameters for TPA. **(E)** Overlaid spectra of the zg30 experiments. The intensity of the TTB internal standard integral has been normalized so that peak intensity is reflective of the change in d1. **(F)** Overlaid spectra of the zg30 experiments. The intensity of the TPA internal standard integral has been normalized so that peak intensity is reflective of the change in d1. RCF conditions: 2 g biomass, 400 mg Ru/C, 225 °C, 30 bar H_2, 3 h, 30 mL 1:1 vol/vol MeOH/H_2O, LLE with ethyl acetate/water. NMR specifications: 0.2 mL of extracted oil dried under flowing N_2 and dissolved in 1 mL 10 acetone-d_6 with ~ 1 mg/mL internal standard. 32 scans, 1-24 s delay (d1), 0.3 Hz line broadening, zero filling to 2x spectrum size.

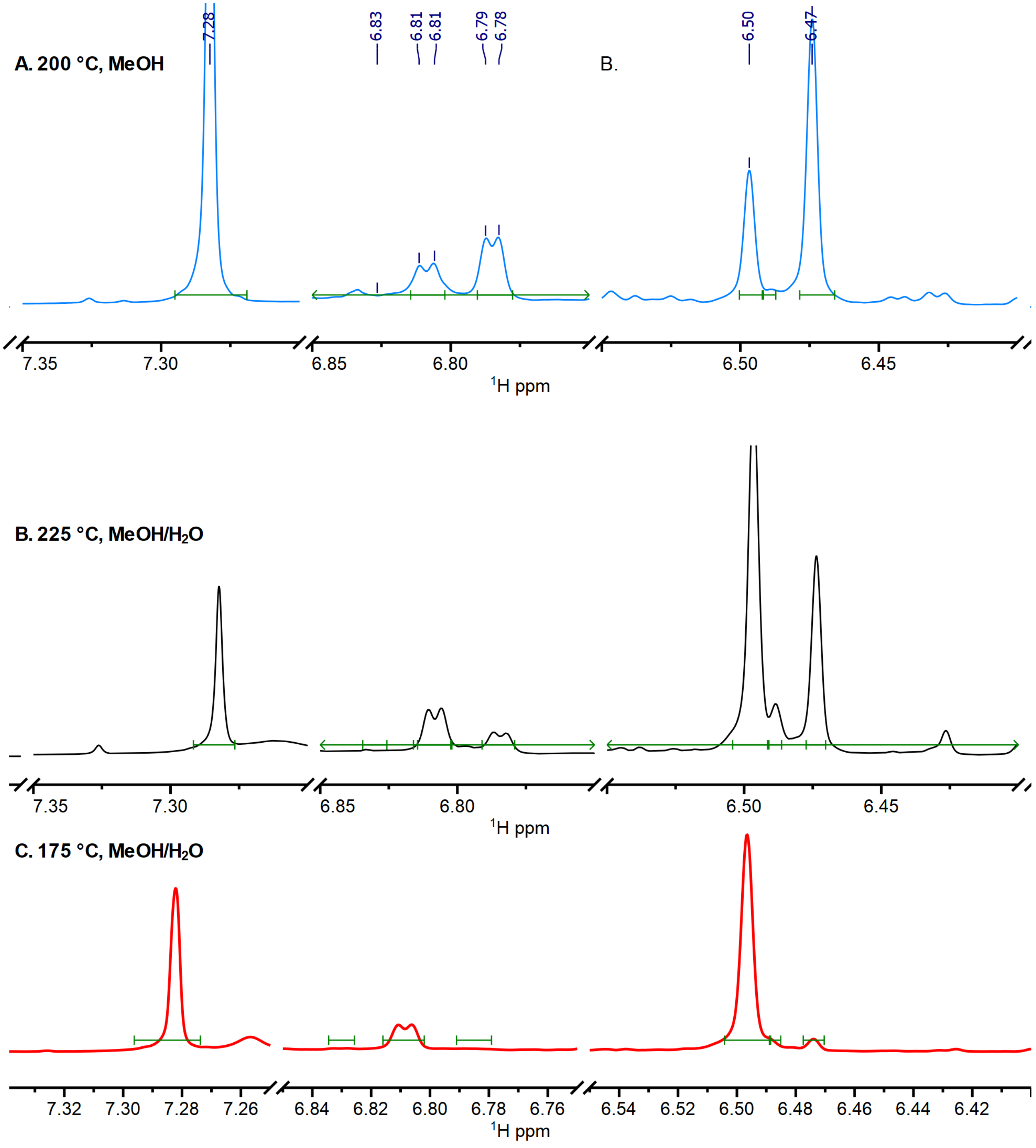

Figure S36. Examples of integration ranges. (A) RCF with methanol as the solvent leading to high selectivity for 4-propyl products (spectrum is the same as presented in **Figure 1A**). **(B)** RCF with 1:1 methanol/water as the solvent at 225 °C leading to intermediate selectivity for 4-propyl and 4-(3-hydroxypropyl) products (spectrum is the same as presented in **Figure 2B**). **(C)** RCF with 1:1 methanol/water as the solvent at 175 °C leading to high selectivity for 4-(3-hydroxypropyl) products (spectrum is the same as presented in **Figure 2C**). RCF conditions: 2 g biomass, 400 mg Ru/C, 30 bar H_2, 3 h, LLE with ethyl acetate/water except for **(A)**. NMR specifications: 0.2 mL of extracted oil dried under flowing N_2 and dissolved in 1 mL 10 acetone-d_6 with ~ 1 mg/mL internal standard. 32 scans, 3 s delay (d1), 0.3 Hz line broadening, zero filling to 2x spectrum size.

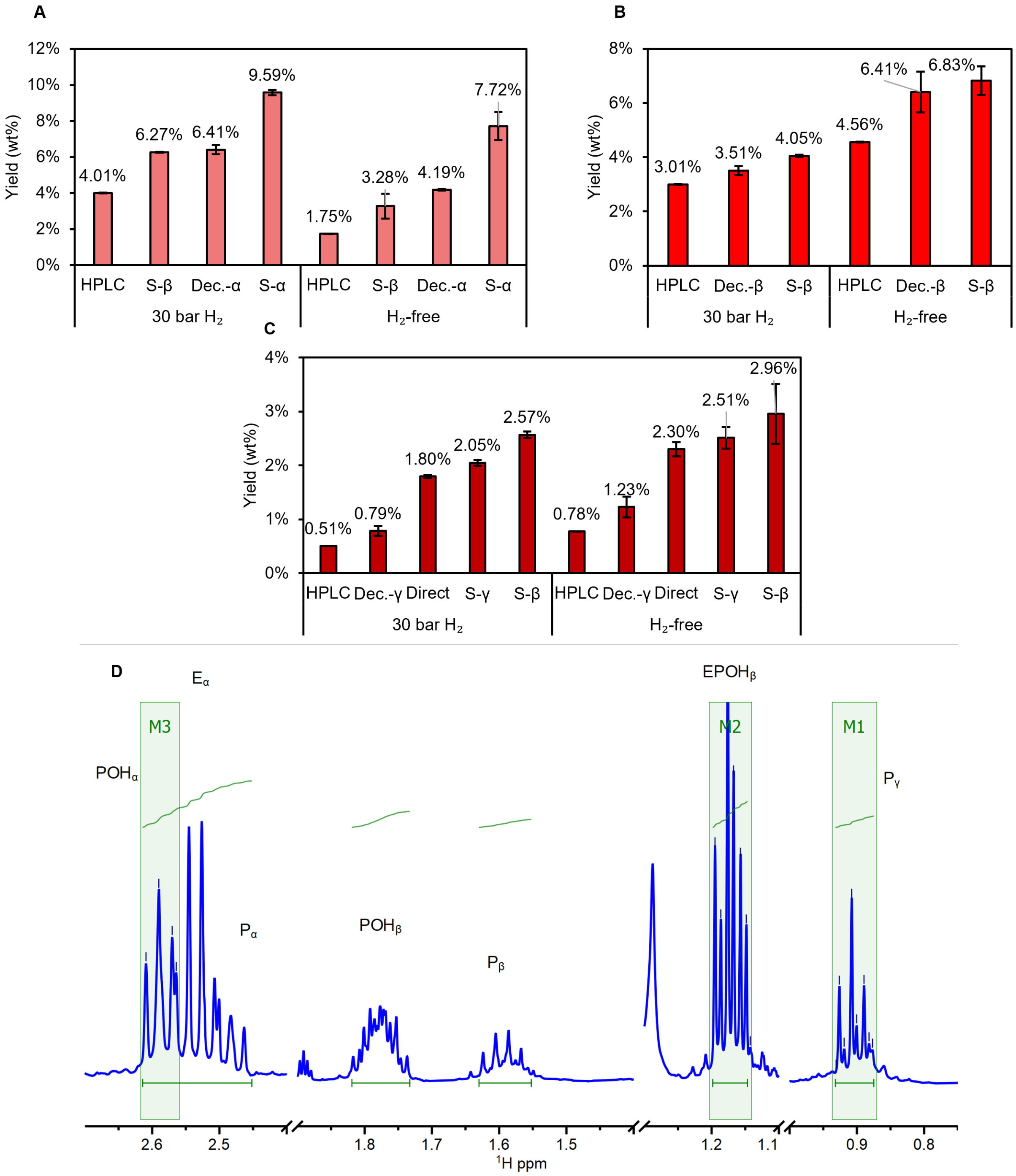

Figure S37. Comparison of methods to quantify guaiacyl monomers from the aliphatic region. (A) 4-(3-hydroxypropyl)-guaiacol. **(B)** 4-ethylguaiacol **(C)** 4-propylguaiacol. Direct means that the area typically integrated was integrated despite unclear/overlapping peaks. S-α/β indicates that the syringyl monomers and other overlapping peaks were subtracted out of the combined aliphatic region. Dec. indicates that the peak was deconvoluted to quantify the guaiacyl monomer using the "Peaks" integral mode in MNova. **(D)** NMR spectrum of RCF oil from Pd/C with 30 bar H_2 leading to intermediate selectivity to 4-(3-hydroxypropyl), 4-propyl, and 4-ethyl side chain products. Green boxes indicate where peak deconvolution was performed.

References

(1) Kenny, J. K.; Neefe, S. R.; Brandner, D. G.; Stone, M. L.; Happs, R. M.; Kumaniaev, I.; Mounfield, W. P.; Harman-Ware, A. E.; Devos, K. M.; Pendergast, T. H.; Medlin, J. W.; Román-Leshkov, Y.; Beckham, G. T. Design and Validation of a High-Throughput Reductive Catalytic Fractionation Method. *JACS Au* **2024**, *4* (6), 2173–2187.

(2) Brandner, D. G.; Kruger, J. S.; Thornburg, N. E.; Facas, G. G.; Kenny, J. K.; Dreiling, R. J.; Morais, A. R. C.; Renders, T.; Cleveland, N. S.; Happs, R. M.; Katahira, R.; Vinzant, T. B.; Wilcox, D. G.; Román-Leshkov, Y.; Beckham, G. T. Flow-through Solvolysis Enables Production of Native-like Lignin from Biomass. *Green Chem.* **2021**, *23* (15), 5437–5441.

(3) Kenny, J. K.; Brandner, D. G.; Neefe, S. R.; Michener, W. E.; Román-Leshkov, Y.; Beckham, G. T.; Medlin, J. W. Catalyst Choice Impacts Aromatic Monomer Yields and Selectivity in Hydrogen-Free Reductive Catalytic Fractionation. *React. Chem. Eng.* **2022**, *7* (12), 2527–2533.

(4) Woodworth, S. P.; Ramirez, K. J.; Beckham, G. T. *Aromatic monomers analysis for reductive catalytic fractionation of lignin by liquid chromatography with diode array detection*. Protocols.io.

(5) Alt, H. M.; Brandner, D. G.; Beckham, G. T.; Ramirez, K. J. *Lignin Reductive Catalytic Fractionation (RCF) Monomers Analysis by Gas Chromatography Flame Ionization Detection (GC-FID)*. Protocols.io.

(6) Rundlöf, T.; Mathiasson, M.; Bekiroglu, S.; Hakkarainen, B.; Bowden, T.; Arvidsson, T. Survey and Qualification of Internal Standards for Quantification by 1H NMR Spectroscopy. *J. Pharm. Biomed. Anal.* **2010**, *52* (5), 645–651.

(7) Bourmaud, C. L.; Bertella, S.; Rico, A. B.; Karlen, S. D.; Ralph, J.; Luterbacher, J. Quantification of Native Lignin Structural Features with Gel-phase 2D-HSQC0 Reveals Lignin Structural Changes during Extraction. *Angew. Chem. Int. Ed.* **2024**, e202404442.
